# scCross: efficient search for rare subpopulations across multiple single-cell samples

**DOI:** 10.1093/bioinformatics/btae371

**Published:** 2024-06-18

**Authors:** Alexander Gerniers, Siegfried Nijssen, Pierre Dupont

**Affiliations:** ICTEAM/INGI/Artificial Intelligence and Algorithms Group, UCLouvain, Louvain-la-Neuve 1348, Belgium; ICTEAM/INGI/Artificial Intelligence and Algorithms Group, UCLouvain, Louvain-la-Neuve 1348, Belgium; ICTEAM/INGI/Artificial Intelligence and Algorithms Group, UCLouvain, Louvain-la-Neuve 1348, Belgium

## Abstract

**Motivation:**

Identifying rare cell types is an important task to capture the heterogeneity of single-cell data, such as scRNA-seq. The widespread availability of such data enables to aggregate multiple samples, corresponding for example to different donors, into the same study. Yet, such aggregated data is often subject to batch effects between samples. Clustering it therefore generally requires the use of data integration methods, which can lead to overcorrection, making the identification of rare cells difficult. We present scCross, a biclustering method identifying rare subpopulations of cells present across multiple single-cell samples. It jointly identifies a group of cells with specific marker genes by relying on a global sum criterion, computed over entire subpopulation of cells, rather than pairwise comparisons between individual cells. This proves robust with respect to the high variability of scRNA-seq data, in particular batch effects.

**Results:**

We show through several case studies that scCross is able to identify rare subpopulations across multiple samples without performing prior data integration. Namely, it identifies a cilium subpopulation with potential new ciliary genes from lung cancer cells, which is not detected by typical alternatives. It also highlights rare subpopulations in human pancreas samples sequenced with different protocols, despite visible shifts in expression levels between batches. We further show that scCross outperforms typical alternatives at identifying a target rare cell type in a controlled experiment with artificially created batch effects. This shows the ability of scCross to efficiently identify rare cell subpopulations characterized by specific genes despite the presence of batch effects.

**Availability and implementation:**

The R and Scala implementation of scCross is freely available on GitHub, at https://github.com/agerniers/scCross/. A snapshot of the code and the data underlying this article are available on Zenodo, at https://zenodo.org/doi/10.5281/zenodo.10471063.

## 1 Introduction

Nowadays, the availability of single-cell omics data is widespread. When designing an experiment, it is common to have access to single-cell data from multiple sources. For instance, one could aggregate single-cell datasets obtained by biopsies of several cancer patients in order to get a better understanding of the pathology. Such larger quantities of data can make it easier to extract information using data-mining techniques, allowing us to gain insights into biological phenomena that could not be retrieved from one single dataset. However, aggregating several single-cell samples comes with pitfalls: batch effects may appear due to the presence of technical variability (differences in sequencing technologies, experimental conditions, etc.) as well as biological variance between donors (Luecken *et al.* 2022). If not handled properly, unsupervised (bi)clustering algorithms could pick up this unwanted variation rather than biologically relevant information.

**Figure 1.**
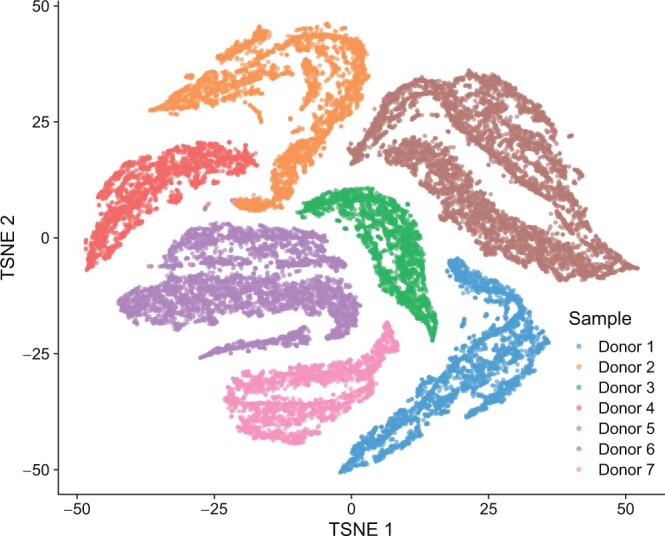
Dimensionality reduction of the NSCLC DTCs dataset using *t*-SNE. Several clusters are visible, but contain cells coming from one single donor. *t*-SNE captures variability between batches rather than grouping cells together by specific expression patterns.

Figure 1 shows the result of *t*-SNE (van der Maaten and Hinton 2008) on a dataset containing 26 027 Non-Small Cell Lung Cancer (NSCLC) Dissociated Tumor Cells (DTCs) coming from seven donors. Several clusters seem to appear in the result. However, when assigning a color based on the origin of each cell, it turns out that the clusters simply identify the donors. The variance of expression captured in this projected and reduced space actually accounts for the batch effects across samples rather than the different cell types present in the data.

To avoid such bias, several data integration techniques have been proposed (Argelaguet *et al.* 2021, Luecken *et al.* 2022). They aim at combining several single-cell samples into one consistent dataset. They generally rely on the definition of anchors that link different datasets. The Mutual Nearest Neighbors (MNNs) method (Haghverdi *et al.* 2018), for instance, identifies pairs of cells in different samples that are mutually nearest to each other, which define the most similar cells of the same type across samples. The difference in expression between MNN pairs, averaged across many such pairs, provides an estimation of the batch effect, from which a correction vector is derived that integrates the different samples. Several other approaches have been build upon this principle, including Seurat v3 (Stuart *et al.* 2019) and Scanorama (Hie *et al.* 2019) which identify MNNs in lower-dimensional representations of the expression data.

While data integration is often necessary to accurately identify cell types in multi-sample single-cell data, improper integration may lead to overcorrection, whereby genuine biological variability is erased along with the batch effects (Büttner *et al.* 2019). This is especially problematic when dealing with rare cells, as improper data integration may make it impossible to detect them (Guo *et al.* 2022). Some methods might also be sensitive to the order in which the samples are integrated, which can lead to spurious alignment between disparate cell types (Hie *et al.* 2019). Moreover, integrating large datasets often requires a high computational cost, both in terms of time and memory, especially for methods performing integration in the original genes × cells space. Methods integrating cells after projection to lower-dimensional spaces tend to be computationally efficient, but at the cost of losing the interpretation in terms of genes and/or cells concerned, making a differential expression analysis impossible. Being able to identify rare cell expression patterns across samples without needing data integration would therefore be useful to analyze the heterogeneity of single-cell data.

In a previous work, we introduce MicroCellClust (Gerniers *et al.* 2021), a method for identifying rare and highly specific subpopulations of cells in single-cell expression data, restricted to one sample (e.g. a single donor sample). This algorithm performs a joint identification of a rare subpopulation of cells and a specific set of marker genes. In other words, it identifies a bicluster of cells and genes. Specifically, it looks for patterns of high expression of certain genes in the cells within such a bicluster, with low expression values for the same genes in the other cells (called *out-of-cluster*). This multivariate approach contrasts with typical alternatives relying on univariate techniques or separate identification of cell types and marker genes. MicroCellClust is based on a global sum criterion, that is the objective value of a bicluster is defined as the sum of the corresponding expression values (Branders *et al.* 2019). This approach proves particularly robust with respect to the high technical and biological variability of single-cell data (Todorov and Saeys 2019). For instance, an unusually high expression of a gene in a particular cell, e.g. due to transcriptional bursting, has little influence since there is no pairwise comparison of this value with those in other cells.

Likewise, the MicroCellClust approach is likely insensitive to batch effects: shifts in expression values between samples have little influence as long as the global value of the bicluster remains high. Even though this approach has been originally tested in a single sample setting it could, in principle, be applied to multiple samples concatenated in a larger expression matrix. Doing so would however not guarantee that MicroCellClust could identify a subpopulation of cells actually shared among different samples (e.g. multiple donors in a cancer study). By design of this original approach, it could simply return a specific subpopulation of cells that would also be restricted to a single donor. In this work we present scCross, an extension to MicroCellClust, in order to identify subpopulations of cells with specific marker genes that are effectively present across multiple samples of single-cell data.

## 2 The scCross method

Let S be a set of single-cell samples (for instance corresponding to different donors). Sample *s* can be represented by a matrix $M_{s}\in R^{\left|G|\times |C_{s}\right|}$, with G the set of rows associated to the genes (assumed identical for each sample) and $C_{s}$ the set of columns associated to the cells. This data can be aggregated in one matrix $M\in R^{\left|G|\times |C\right|}$, with $C=\underset{s\in S}{\cup }C_{s}$, by concatenating the data of each sample: $M=(M_{1}\;\cdots \;M_{\left|S\right|})$. An entry *m_ij_* of this matrix is here assumed positive only if cell *j* expresses gene *i*, and negative otherwise [The (normalized) count data are here log-transformed using ${\;\text{log}\;}_{10}(x+0.1)$, where the 0.1 pseudocount yields a −1 value for zero-counts, while (normalized) counts ≥0.9 remain positive.].

The goal is to select a bicluster, i.e. a subset of genes I⊆G and a subset of cells J⊆C representative of a small subpopulation of cells with specific genes. A gene *i* is considered specific whenever it is highly expressed for the cells in *J*, and has low expression in the cells not included in *J*. Considering one sample *s*, the specificity of gene *i* w.r.t. the selected cells within this sample is measured by:

(1) $$\omega_{is}(J)=\sum_{j\in C_{s}\cap J}m_{ij}-\kappa \sum_{k\in C_{s}\setminus J}\max\{0,m_{ik}\}.$$

The first term sums up the expression values corresponding to the selected cells within this sample ($j\in C_{s}\cap J$) to assess whether they highly express gene *i*. The second term penalizes any positive expression in the remaining cells from that same sample ($k\in C_{s}\setminus J$), to assess whether expression of gene *i* is indeed specific to the selected cells. The relative influence of this penalty is controlled by the *κ* parameter (default: $\kappa =\frac{100}{\left|C\right|}$). The higher this value, the fewer genes are selected in *I* as only the most differentiated ones have a positive *ω* value.

Computing an optimal bicluster $\left(I^{*},J^{*}\right)$ is performed through solving the following optimization problem:

(2) $$(I^{*},J^{*})=\underset{\overset{I\subseteq G}{J\subseteq C}}{\text{argmax}}\sum_{i\in I}\delta_{i}(J)\sum_{s\in S}\omega_{is}(J).$$

(3) $$\text{such that}\;\;\frac{\left|\{(i,j)|i\in I,j\in J,m_{ij}<0\}\right|}{\left|I|\cdot |J\right|}\leq \mu .$$

The objective function in the Equation (2) sums the *ω* values for each gene and each sample. This global sum allows for variations within the expression values, as they are not compared in a pairwise fashion, which is well suited to the technical and biological variability of scRNA-seq data. The parameter *μ* in the constraint (3) controls the proportion of negative values allowed in the solution (by default 10%) to ensure that the selected genes are sufficiently often expressed across the bicluster. Without such a constraint, many negative values tend to be included, leading to a result containing many cells with low similarity between them (Gerniers *et al.* 2021). Furthermore, the M matrix is assumed to be sparse in terms of positive expression values. Genes with positive values for many (or even all) cells represent generic markers of high expression throughout the cell population instead of rare expression patterns we are after. Such genes are removed from the matrix M and therefore from the set G of genes to be considered [One typically discards genes expressed in strictly more than 25% of the cells, which is consistent with the global sum criterion used in Equation (2), which leads to select highly expressed genes in the selected cells. Yet, it is possible to search for other types of patterns by appropriately preprocessing the data (we stick here for clarity to the original interpretation with selected entries corresponding to high expression). See Section D of the Supplementary Data for an analysis of the impact of this threshold on the initial set of genes.).

The $\delta_{i}(J)$ factor is intended to evaluate the similarity between the expression of a gene *i* across the different samples. Without it, i.e. setting it to 1, the objective (2) is equivalent to the original MicroCellClust optimization problem (Gerniers *et al.* 2021). Indeed, simply summing the gene contributions of each sample ($\sum_{i}\sum_{s}\omega_{is}$) amounts to computing the gene contributions over the entire dataset ($\sum_{i}\omega_{i}$), thus ignoring the partition into different samples. One could therefore identify a subpopulation that is specific to one sample, but not observed in others.

The $\delta_{i}(J)$ term penalizes genes that are not expressed similarly among all samples, i.e. with large differences between the *ω_is_* of gene *i*. This factor is defined as the ratio between the geometric mean [i.e. the *n*th root of the product of *n* quantities (Negative *ω_is_* are replaced by some small positive constant ε>0, e.g. 0.1, to stay in the real domain even when *n* is pair.)] and the arithmetic mean:

(4) $$\delta_{i}(J)=\frac{{\left(\prod_{s\in S}\max\{\varepsilon ,\omega_{is}(J)\}\right)}^{\frac{1}{\left|S\right|}}}{\frac{1}{\left|S\right|}\sum_{s\in S}\max\{\varepsilon ,\omega_{is}(J)\}}.$$

The geometric mean has a high value, close to the arithmetic mean, when gene *i* is expressed similarly across all samples (if all *ω_is_* tend to be equal then *δ_i_* tends to 1 and no penalty is applied), but would be lower than the arithmetic mean in case of differences between the *ω_is_* values. In the later case, *δ_i_* induces an effective penalty and lowers the influence of the gene *i* in the global objective to be maximized. This weighting thus gives a comparative advantage to genes that are present across all (or most) samples, and cancels out those specific to only one (or a few) sample(s).

The choice of the proposed *δ* metric is further motivated by the fact that the geometric mean is more influenced by higher values. A gene with high expression values in all but a few samples would only be slightly penalized. In contrast, a gene that is highly expressed in only a few samples gets a heavy penalty (i.e. a low *δ* value), even though alternative metrics such as the variance may be equal in both cases.

**Figure 2.**
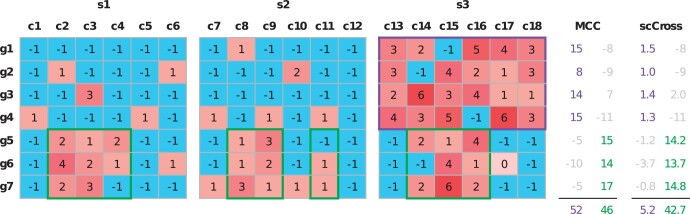
Toy example comparing the results of MicroCellClust (MCC) and scCross for two distinct subpopulations. The purple bicluster is the optimal MicroCellClust solution (with *κ *= 1 and μ=0.1). It corresponds to the subpopulation $J_{1}=\{13,14,15,16,17,18\}$, which is specific to the third sample. The green bicluster is the optimal scCross solution (with same *κ* and *μ* values). It corresponds to the subpopulation $J_{2}=\{2,3,4,8,9,11,14,15,16\}$, distributed over the three samples. The numbers on the right indicate the contribution of each gene under the two different optimization objectives, with the final objective value of the bicluster below. Numbers in gray indicate that the corresponding genes are not selected, either because their contribution is negative or because they violate the constraint (3).

In summary, scCross improves upon MicroCellClust to analyze multi-sample data by evaluating each sample separately ($\sum_{s\in S}^{\;}$) and by introducing a regularization factor between them (*δ_i_*). Figure 2 shows the evaluation of both objectives on a toy example. The purple bicluster has the maximal value under the MicroCellClust objective function. In contrast, its value vanishes according to the scCross objective as indeed the selected bicluster is restricted only to cells from the third sample. The green bicluster is the scCross optimal solution and includes some cells from all samples.

**Figure 3.**
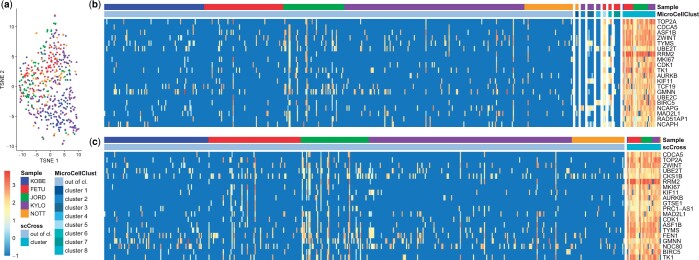
Breast cancer GARP+ Tregs from five donors. (a) Dimensionality reduction using *t*-SNE. No distinct clusters emerge, which indicates this data seems homogeneous and does not suffer from the presence of batch effects. (b) Result of eight runs of MicroCellClust. The genes displayed are those corresponding to the eighth cluster (only the top 20 with highest contribution to the objective are shown). These genes are highly expressed in cluster 8, but also in cluster 5, which has been identified earlier and grouped to sample-specific genes. (c) The result of scCross showing the 20 marker genes with highest contribution to its objective. The cluster 8 of MicroCellClust is identified during the first run, and includes the three cells from cluster 5.

Despite the NP-hard nature of the scCross optimization problem, a good approximation is found in linear time w.r.t. the number of cells using an adaptation of the MicroCellClust 2 solver (Gerniers and Dupont 2022). It is based on a beam search, but with different beams for each sample to ensure the diversity of the search (see the Supplementary Material for details). One such run only identifies one bicluster, leaving the remaining cells unclustered (or *out-of-cluster*). Yet, one can easily identify other biclusters by running scCross again while excluding already selected cells from the pool of candidate variables. These cells can no longer be selected in *J*, but their expression can still have an influence on the second term of the objective (1) which counts out of cluster expression (Alternatively, one could opt to exclude previously identified genes to identify new biclusters. Such a strategy might be useful to identify hierarchical structures as cells might be clustered multiple times. In this work, we stick to the first option to produce the results described in Figs 3, 5, and 6.). In order to decide the number of runs to perform, we suggest to validate biologically the results of each run (e.g. using a Gene Ontology analysis Ashburner *et al.* 2000) and to stop whenever additional runs seem to return uninteresting results (typically clusters of two cells with genes not linked to any GO term, suggesting that all rare subpopulations have already been found).

## 3 Results

We show through multiple experiments on scRNA-seq data that scCross is capable of identifying common subpopulations across multiple samples, without the need for data integration. First, we show that scCross increases the quality of the solution compared to MicroCellClust. Then, we show that scCross outperforms typical alternatives in a controlled experiment with artificial batch effects, where a specific rare cell type serves as target. We further show that scCross is robust with respect to data integration as it identifies (i) a cilium subpopulation within bone marrow sequenced from seven donors, and (ii) several subpopulations from eight pancreas samples sequenced using two different technologies. Finally, we show that scCross can be used in a different setting, namely, to search for rare cells across different organs, here tested on mouse data.

### 3.1 scCross identifies common cell-cycle genes across GARP+ Treg samples

In a previous study (Gerniers *et al.* 2021), MicroCellClust has been used to analyze a sample of GARP+ Tregs from a breast tumor, which revealed a subpopulation with marker genes linked to cell division functions. Here, we extend this study by aggregating the data from five different donors. All samples are sequenced using the Smart-seq2 protocol (Picelli *et al.* 2014), normalized to counts per million and ${\;\text{log}\;}_{10}(x+0.1)$ transformed, for a total of 505 cells, and 19 722 genes expressed in at most 25% of the cells. Interestingly, a *t*-SNE dimensionality reduction (Fig. 3a) does not show any evidence of batch effects between these five samples.

Figure 3b shows the result of multiple MicroCellClust runs, whereby cells identified in previous runs are left out in order to search for a new bicluster in the remaining cells. MicroCellClust first identifies a series of small, donor-specific, subpopulations. It is only during the eighth run that it identifies a subpopulation with 31 cells shared between multiple donors, which exhibits 55 cell-cycle related genes. Moreover, the cells from cluster 5 seem related to this subpopulation as they also express these same genes. Yet, they were clustered beforehand base on a series of donor-specific genes, for which a Gene Ontology (GO) analysis (Ashburner *et al.* 2000) did not yield any function (with a significance threshold of 0.05 including FDR correction). This shows that cells might be classified based on sample-specific artifacts in the absence of an objective function targeted to cross-sample results.

This is not the case with scCross as it identifies this subpopulation directly (Fig. 3c), including the three cells from cluster 5 (for a total of 31 cells and 60 genes). Interestingly, this new cell cluster has a 10% higher value under the MicroCellClust objective than cluster 8, showing that successive runs can prevent MicroCellClust from reaching the local optimum. Moreover, scCross identifies a cell expressing these cell-cycle genes within the NOTT sample. This is not identified with MicroCellClust, showing scCross is able to identify related cells across samples even if present in very small amounts. This illustrates that scCross outperforms MicroCellClust at identifying subpopulations shared across multiple samples.

### 3.2 scCross outperforms alternatives in a controlled Jurkat v. 293 T experiment

To assess the performance of scCross as an alternative to the usual data integration + clustering approach, we designed a controlled experiment using a dataset containing two human immortalized cell lines: 293 T and Jurkat cells (Zheng *et al.* 2017a, Zheng *et al.* 2017b). Several rare populations of Jurkat cells (containing 6, 12, or 20 cells; with 10 independent sampling runs for each size) are sampled at random from the 1694 available ones. For each one, an abundant population of 293 T cells is sampled from the 1540 available ones to obtain mixed datasets of 1000 cells, with ≈15 000 genes (≈10 800 of which are expressed in at most 25% of the cells and are used by scCross).

To simulate batch effects, each dataset is randomly divided in two samples of 500 cells (each one containing the same number of rare Jurkat cells). The first sample is left untouched, whereas the expression values of the second one are multiplied by some value to artificially create batch effects. Three different scenarios are used: “Global” roughly divides the expression values of each gene by two (i.e. each gene is multiplied by a random value generated by a Gaussian distribution with μ=0.5 and σ=0.02); “Gene-specific” multiplies all values of the same gene by a random constant (generated by a uniform distribution in [0.2,1.8], which differs for each gene); “Value-specific” multiplies each expression value independently by a different constant (generated by a uniform distribution in [0.5,1.5]). The ability to identify the rare Jurkat cells is measured by the *F*_1_ score, i.e. the harmonic mean between precision (the proportion of Jurkat cells within the solution) and recall (the proportion of Jurkat cells to be found actually included in the solution).

scCross is compared to three rare cell clustering methods used in conjunction with data integration. RaceID3 (Herman *et al.* 2018) first performs a regular clustering and then identifies outliers to define a new partition that pays attention to rare cells. GiniClust3 (Dong and Yuan 2020) first identifies *high Gini genes*, i.e. genes that are differentially expressed in a limited number of cells, and then performs a clustering restricted to these genes. The scAIDE method (Xie *et al.* 2020) relies on an autoencoder to embed the genes into 256 dimensions, in which an RPH-kmeans clustering is performed.

For each of these methods, the data is first integrated using MNN (Haghverdi *et al.* 2018), Seurat v3 (Stuart *et al.* 2019), or Scanorama (Hie *et al.* 2019). This choice of methods is motivated by the fact they return corrected counts, i.e. do not embed the data in lower dimensional spaces but conserve the gene × cell space, and do not use any additional prior information such as cell type labels, making them usable in the unsupervised setting that is (bi)clustering. In particular, Scanorama is the only top performing method from the Luecken *et al.* (2022) benchmark that satisfies these two requirements.

Table 1 reports the average *F*_1_ over 10 runs for scCross and each combination of data integration + clustering. scCross consistently achieves a high F1 score (≥90%), making it the best performing approach in six out of the nine cases. Moreover, the result of scCross seems robust w.r.t. the different batch effects, as the difference in *F*_1_ between the three scenarios for the same number of rare cells is at most 2%.

**Table 1.** Identification results of rare Jurkat cells among 293 T cells with artificially created batch effects: average *F*_1_ score over 10 independent runs.

Nb. rare	Batch type	scCross	MNN			Seurat v3			Scanorama		
			RaceID3	GiniClust3	scAIDE	RaceID3	GiniClust3	scAIDE	RaceID3	GiniClust3	scAIDE
20	Global	0.95	0.79	0.83	0.14	0.71	**1.00**	0.10	0.63	0.22	0.53
20	Gene-specific	**0.97**	**0.98**	0.46	0.23	0.52	0.47	0.16	0.84	0.43	0.51
20	Value-specific	0.96	0.98	0.87	0.07	0.16	0.31	**1.00**	0.35	0.66	0.39
12	Global	**0.92**	0.74	0.22	0.07	0.63	0.68	0.06	0.37	0.05	0.14
12	Gene-specific	**0.90**	0.72	0.32	0.21	0.47	0.34	0.08	0.46	0.10	0.26
12	Value-specific	0.92	0.82	0.81	0.05	0.12	0.47	**0.95**	0.37	0.08	0.14
6	Global	**0.92**	0.50	0.07	0.03	0.55	0.59	0.09	0.39	0.12	0.03
6	Gene-specific	**0.92**	0.56	0.14	0.06	0.60	0.62	0.09	0.23	0.04	0.05
6	Value-specific	**0.93**	0.80	0.26	0.02	0.60	0.59	0.20	0.35	0.08	0.05

*Note*: Bold results indicate which method outperforms the others for each experiment according to a paired t-test with significance threshold of 0.05 (multiple bold values for the same experiment indicates there is no significant difference between them).

This is not the case for the other approaches. First, one observes the *F*_1_ score of the different clustering methods are dependent on the integration method: RaceID3 generally achieves a higher *F*_1_ with MNN, whereas GiniClust3 overall performs better with Seurat v3. scAIDE, on the other hand, reaches a higher *F*_1_ with Seurat v3 in half of the cases, and with Scanorama in the other half.

Moreover, when comparing the result of a given clustering method between the three scenarios, one observes large differences in *F*_1_. This shows that data integration methods, while aiming at correcting the batch effects, are not always able to reconstitute the original data. Consequently, the differences in expression values in the resulting datasets lead to varying clustering performances. The most striking example is given by scAIDE, which achieves relatively high *F*_1_ scores of 0.98 and 0.76 on the original data with 20 and 12 rare cells, respectively. Yet, this performance vanishes in nearly all cases once batch effects are introduced and subsequently corrected by the data integration methods. This case study thus shows a concrete example where data integration fails to accurately compensate for batch effects.

One further observes that whenever a clustering method outperforms scCross for a particular type of batch effect, its performance is lower on the same data transformed differently. This is for instance the case for scAIDE, which achieves a perfect performance when applied, after integration with Seurat v3, on the 20-jurkat datasets with “value-specific” perturbations. Yet, its performance crashes when used on the same data but with different types of batch effects. In a truly unsupervised single-cell experiment, there would be no way to monitor in which situation, and with which data integration method, a particular clustering method would perform well. This demonstrates the benefit of using scCross, which consistently identifies the same subpopulation of cells regardless of the nature of the batch effects present in the data.

### 3.3 scCross identifies a cilium subpopulation across different donors

**Figure 4.**
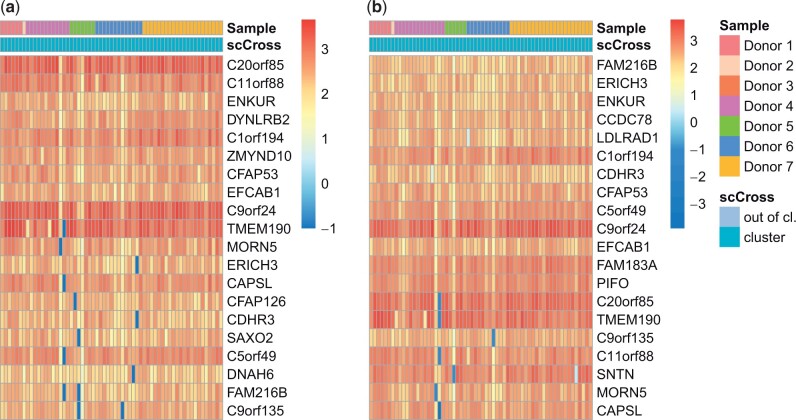
Result of scCross on the NSCLC DTCs dataset. Using (a) the original data, and (b) the integrated data. For each one, the 20 first genes are displayed. Note that out-of-cluster cells are not displayed. See the Supplementary Material for full heatmaps.

Next, we use scCross to analyze the 26 027 NSCLC DTCs dataset from 10× Genomics (https://www.10xgenomics.com/resources/datasets), in which we observed strong batch effects between the seven donors (Fig. 1). This data contains 31 094 genes, 27 452 of which are expressed in at most than 25% of the cells. The scCross result consists of 61 cells, coming from six out of the seven donors (Fig. 4a). This subpopulation is characterized by 77 genes [Using κ=0.2 and μ=0.1. The value of *κ* has been raised from its default value of 0.004 so as to keep only the most differentiated genes (see details in the Supplementary Material)]. A GO analysis (see the Supplementary Material for details) links them to the cilium, an organelle whose dysfunction causes various genetic diseases. Moreover, many of these genes have been identified in CiliaCarta (Van Dam *et al.* 2019), a compendium of ciliary genes (see the Supplementary Material for details). Yet, 33 of the genes identified by scCross are not listed in CiliaCarta, even though the scCross result shows they appear to be strongly related to know ciliary genes. Since several hundred proteins are thought to be involved in ciliary functions and identifying new ones is still ongoing, this observation is definitively worth further investigation.

Interestingly, typical rare cell clustering methods do not identify this cilium subpopulation. RaceID3 scatters these cells in 13 different clusters. To evaluate whether using data integration could enable RaceID3 to identify this cluster, we use MNN to integrate the samples of the seven donors. Yet, the cilium subpopulation is still divided among several (here, 15) clusters.

GiniClust3 clusters cells by first identifying *high Gini genes*. Yet, none of the genes identified by scCross are labeled as such. These cells are thus missed by GiniClust3 and grouped within a large cluster of 2018 cells. Using the MNN-integrated data does not improve this result, since again none of these genes are labeled as *high Gini* and the identified cells are scattered in 14 different clusters.

Finally, it appears that the lower dimension projection used by scAIDE is not effective to capture the cilium related genes, as the corresponding cells are scattered in different larger clusters (both with the original and integrated data). These comparisons further illustrate the ability of scCross to identify specific subpopulations that are missed by typical alternatives, even when used in conjunction with data integration.

sscCross has also been applied on integrated data to evaluate its robustness with respect to batch effects (Fig. 4b). This run results in a subpopulation of 61 cells (57 in common with the previous result, i.e. 93% of the identified cells) and 79 genes (73 in common with the result without data integration). This illustrates that scCross identifies the same cilium linked subpopulation whether or not the data is integrated.

### 3.4 scCross identifies subpopulations in pancreas data from two studies

**Figure 5.**
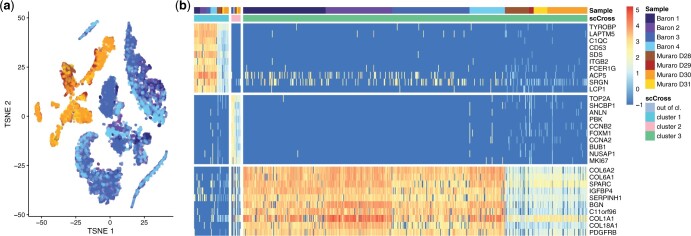
Results of the pancreas case study. (a) Dimensionality reduction of the pancreas data using *t*-SNE. One observes strong batch effects between the data from the two different studies. (b) Result of scCross with 10 genes displayed for each cluster (out-of-cluster cells, which represent 96% of the total, are not displayed). One observes a significant shift in expression levels between the cells of the two studies. Yet, the global sum criterion used in scCross ensures related cells are grouped into the same cluster.

We aggregate eight samples of the human pancreas from cadaveric donors, sequenced with different technologies in two studies. Baron *et al.* (2016a, 2016b) have sequenced four samples containing respectively 1937, 1724, 3605, and 1303 cells using the inDrop protocol (Klein *et al.* 2015). Muraro *et al.* (2016a, 2016b) have sequenced 4 samples of 768 cells each with the CEL-seq2 protocol (Hashimshony *et al.* 2016). There are 16 251 genes in common between the two studies (respectively 82% and 86% of the genes returned by inDrop and CEL-seq2), 13 055 of them are used by scCross as they are expressed in at most 25% of the cells. A *t*-SNE dimensional reduction (Fig. 5a) reveals that there are strong batch effects between the two studies, as all clusters exclusively contain cells from the same study. Yet, one expects to find shared expression patterns across samples, corresponding to cell types linked to the function of the pancreas in the human body, beyond sample-specific patterns coming from these batch effects.

Figure 5b shows the result of scCross. First, it identifies a subpopulation of 53 cells characterized by 22 genes linked to an immune response (according to a GO analysis, see the Supplementary Material). Interestingly, these cells are well distributed across the eight donors even though there is a visible difference in expression between the samples of the two studies. This visible difference is probably due to the differences in the sequencing pipeline between the two studies. When performing further runs of scCross, additional subpopulations shared by all eight donors are exhibited. They consist of a tiny subpopulation of 14 cells with 37 cell cycle genes and a subpopulation of 526 cells with 17 genes involved in collagen and structural organization. These results demonstrate the ability of scCross to effectively group related cells together despite strong differences in expression levels induced by batch effects.

### 3.5 scCross identifies rare subpopulations across different mouse organs

**Figure 6.**
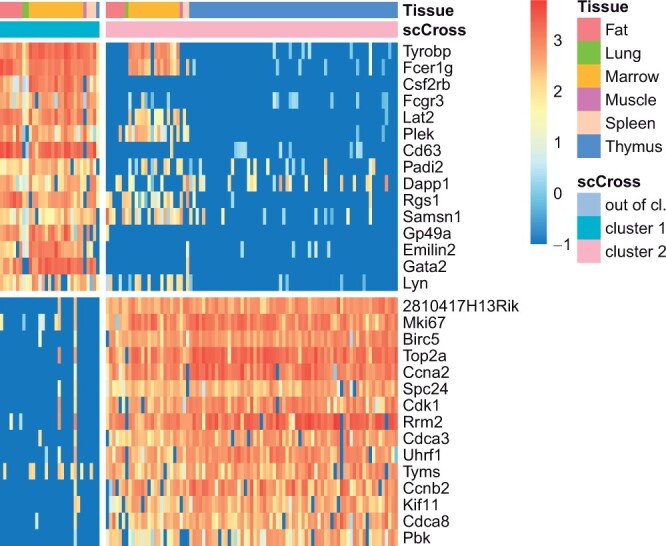
Result of scCross on the *Tabula Muris* data. For each cluster, the 15 best genes are displayed (out-of-cluster cells, i.e. 96% of the total, are not displayed).

Rather than searching for shared rare expression patterns across different donors, scCross can also be used to find rare subpopulations present across other types of samples, such as different organs. The *Tabula Muris* (Tabula Muris Consortium 2018a, Tabula Muris Consortium 2018b) let us compare gene expression in cell types that are shared between distinct tissues. More specifically, scCross has been used to analyze 2251 T cells sequenced across six organs using the Smart-seq2 protocol (Fig. 6), with 15 024 genes expressed in at most 25% of the cells. The first run results in a subpopulation of 31 cells characterized by 61 genes. A GO analysis links these genes to leukocyte activation. Unsurprisingly, the majority of the cells come from the bone marrow, where leukocytes are formed from stem cells. Yet, seven cells also come from the fat, as well as a few cells in other tissues, which could constitute a sign of inflammation.

A second run of scCross finds a cluster of 91 cells with 57 genes indicating proliferation (including Top2a, Rrm2, Kif11, and Pbk). A part of this cluster has been identified in the original publication related to this data (Tabula Muris Consortium 2018), but it was interpreted as a cluster of thymic T cells. Even though the large majority of the cells indeed come from the thymus, scCross shows proliferation also appears in the bone marrow and fat, as well as a few cells in the lung, muscle and spleen. This illustrates that scCross can be used to analyze rare cell types across organs.

## 4 Conclusion

We propose scCross, a method for identifying small subpopulations of cells with specific genes across multiple single-cell samples. It relies on a global sum criterion, which proves insensitive to the batch effects typically present in multi-sample data. Moreover, it explicitly searches for shared expression patterns across samples, which contrasts with the traditional two-step approach of first integrating datasets (i.e. transforming the data into one sample) and then using traditional clustering algorithms (thus ignoring the partition into multiple samples).

The reported experiments show scCross effectively groups related cells together, even with strong batch effects resulting from the use of different sequencing technologies. These experiments also show the ability of scCross to jointly identify specific genes for each subpopulation. In particular, a case study on lung cancer cells show that it identifies a subpopulation characterized by known ciliary genes, together with other genes that have not yet been linked to the cilium. This particular subpopulation is missed by typical alternatives, even when they run on integrated data. Moreover, scCross shows a higher stability than alternative methods on a controlled experiment where different kind of batch effects are artificially created. Such result shows the effectiveness of scCross for a joint identification of cells and genes.

The main focus of scCross is to identify subpopulations shared by different donors in single-cell data but it can be used in other research settings. We show that it can also be used, for example, to identify common genes across different organs. Moreover, the underlying optimization problem is generic: it only takes as input a gene × cell data matrix, and a vector indicating to which sample each cell belongs. It therefore makes no assumption on how the different samples are generated, and could even be applied to other data types than scRNA-seq.

In this paper, we focus on data coming from different samples where the same modality is sequenced (here scRNA-seq), also called *horizontal* integration. It could be interesting to adapt this method to deal with *vertical* integration, whereby several modalities are sequenced for the same sample of cells, in order to find subpopulations with strong links between the different modalities. This aspect will be part of our future research.

## Supplementary Material

btae371_Supplementary_Data

## References

[btae371-B1] Argelaguet R , Cuomo ASE, Stegle O et al Computational principles and challenges in single-cell data integration. Nat Biotechnol 2021;39:1202–15. 33941931 10.1038/s41587-021-00895-7

[btae371-B2] Ashburner M , Ball CA, Blake JA et al Gene ontology: tool for the unification of biology. Nat Genet 2000;25:25–9. 10802651 10.1038/75556 PMC3037419

[btae371-B3] Baron M , Veres A, Wolock SL et al A single-cell transcriptomic map of the human and mouse pancreas reveals inter-and intra-cell population structure. Cell Syst2016a;3:346–60.e4. 27667365 10.1016/j.cels.2016.08.011 PMC5228327

[btae371-B4] Baron M , Veres A, Wolock SL et al A single-cell transcriptomic map of the human and mouse pancreas reveals inter-and intra-cell population structure. Gene Expression Omnibus2016b;GSE84133. 10.1016/j.cels.2016.08.011 PMC5228327 27667365

[btae371-B5] Branders V , Schaus P, Dupont P et al Identifying gene-specific subgroups: an alternative to biclustering. BMC Bioinformatics 2019;20:625. 31795929 10.1186/s12859-019-3289-0 PMC6888937

[btae371-B6] Büttner M , Miao Z, Wolf FA et al A test metric for assessing single-cell RNA-seq batch correction. Nat Methods 2019;16:43–9. 30573817 10.1038/s41592-018-0254-1

[btae371-B7] Dong R , Yuan G-C. GiniClust3: a fast and memory-efficient tool for rare cell type identification. BMC Bioinformatics 2020;21:158. 32334526 10.1186/s12859-020-3482-1 PMC7183612

[btae371-B8] Gerniers A , Dupont P. MicroCellClust 2: a hybrid approach for multivariate rare cell mining in large-scale single-cell data. In: *2022 IEEE International Conference on Bioinformatics and Biomedicine (BIBM)*, Las Vegas, NV, USA: IEEE Computer Society, pp. 148–53. 2022.

[btae371-B9] Gerniers A , Bricard O, Dupont P et al MicroCellClust: mining rare and highly specific subpopulations from single-cell expression data. Bioinformatics 2021;37:3220–7. 33830183 10.1093/bioinformatics/btab239 PMC8504615

[btae371-B10] Guo T , Chen Y, Shi M et al Integration of single cell data by disentangled representation learning. Nucleic Acids Res 2022;50:e8. 34850092 10.1093/nar/gkab978 PMC8788944

[btae371-B11] Haghverdi L , Lun ATL, Morgan MD et al Batch effects in single-cell RNA-sequencing data are corrected by matching mutual nearest neighbors. Nat Biotechnol 2018;36:421–7. 29608177 10.1038/nbt.4091 PMC6152897

[btae371-B12] Hashimshony T , Senderovich N, Avital G et al CEL-Seq2: sensitive highly-multiplexed single-cell RNA-seq. Genome Biol 2016;17:77. 27121950 10.1186/s13059-016-0938-8 PMC4848782

[btae371-B13] Herman JS , Grün D, Sagar et al FateID infers cell fate bias in multipotent progenitors from single-cell RNA-seq data. Nat Methods 2018;15:379–86. 29630061 10.1038/nmeth.4662

[btae371-B14] Hie B , Bryson B, Berger B et al Efficient integration of heterogeneous single-cell transcriptomes using Scanorama. Nat Biotechnol 2019;37:685–91. 31061482 10.1038/s41587-019-0113-3 PMC6551256

[btae371-B15] Klein AM , Mazutis L, Akartuna I et al Droplet barcoding for single-cell transcriptomics applied to embryonic stem cells. Cell 2015;161:1187–201. 26000487 10.1016/j.cell.2015.04.044 PMC4441768

[btae371-B16] Luecken MD , Büttner M, Chaichoompu K et al Benchmarking atlas-level data integration in single-cell genomics. Nat Methods 2022;19:41–50. 34949812 10.1038/s41592-021-01336-8 PMC8748196

[btae371-B17] Muraro MJ , Dharmadhikari G, Grün D et al A single-cell transcriptome atlas of the human pancreas. Cell Syst2016a;3:385–94.e3. 27693023 10.1016/j.cels.2016.09.002 PMC5092539

[btae371-B18] Muraro M , Dharmadhikari G, Grün D et al A single-cell transcriptome atlas of the human pancreas. Gene Expression Omnibus2016b;GSE85241. 10.1016/j.cels.2016.09.002 PMC5092539 27693023

[btae371-B19] Picelli S , Faridani OR, Björklund AK et al Full-length RNA-seq from single cells using Smart-seq2. Nat Protoc 2014;9:171–81. 24385147 10.1038/nprot.2014.006

[btae371-B20] Stuart T , Butler A, Hoffman P et al Comprehensive integration of single-cell data. Cell 2019;177:1888–902.e21. 31178118 10.1016/j.cell.2019.05.031 PMC6687398

[btae371-B22] Tabula Muris Consortium. Single-cell transcriptomics of 20 mouse organs creates a Tabula Muris. Nature2018a;562:367–72. 30283141 10.1038/s41586-018-0590-4 PMC6642641

[btae371-B21] Tabula Muris Consortium. Single-cell RNA-seq data from Smart-seq2 sequencing of FACS sorted cells (v2). Figshare 2018b, 5829687.v7.

[btae371-B23] Todorov H , Saeys Y. Computational approaches for high-throughput single-cell data analysis. FEBS J 2019;286:1451–67. 30058136 10.1111/febs.14613

[btae371-B24] van Dam TJP , Kennedy J, van der Lee R et al CiliaCarta: an integrated and validated compendium of ciliary genes. PLoS One 2019;14:e0216705. 31095607 10.1371/journal.pone.0216705 PMC6522010

[btae371-B25] van der Maaten L , Hinton G. Visualizing data using t-SNE. J Mach Learn Res 2008;9:2579–605.

[btae371-B26] Xie K , Huang Y, Zeng F et al scAIDE: clustering of large-scale single-cell RNA-seq data reveals putative and rare cell types. NAR Genom Bioinform 2020;2:lqaa082. 33575628 10.1093/nargab/lqaa082 PMC7671411

[btae371-B28] Zheng GXY , Terry JM, Belgrader P et al Massively parallel digital transcriptional profiling of single cells. Nat Commun2017a;8:14049. 28091601 10.1038/ncomms14049 PMC5241818

[btae371-B27] Zheng GXY , Terry JM, Belgrader P et al 50%:50% jurkat:293T cell mixture. Sequence Read Archive, 2017b;SRX1723923.

