# Supplementary material for: scCross: efficient search for rare subpopulations across multiple single-cell samples
Source: Bioinformatics. 2024 Jun 18;40(6):btae371. doi: 10.1093/bioinformatics/btae371 (PMC11256925; doi:10.1093/bioinformatics/btae371)
Supplement: btae371_Supplementary_Data [file btae371_supplementary_data.pdf]

scCross: efficient search for rare subpopulations across multiple single-cell samples

Supplementary material

A. Gerniers, S. Nijssen and P. Dupont

A Breast cancer data description

The data used for the first case study is composed of T lymphocytes extracted from 5 human breast tumors. These cells are sorted in a 384-well plate by flow cytometry in order to separate activated regulatory T cells (CD3+ CD4+ CD25+ GARP+) from other phenotypes. This results in a total of 505 cells across 5 samples (KOBE: 96 cells; FETU: 96 cells; JORD: 72 cells; KYLO: 192 cells; NOTT: 49 cells). These cells are processed following the Smart-seq2 protocol to generate scRNA-seq data on the Illumina HiSeq4000 platform. The cleanhop algorithm (`https://rdrr.io/github/obricard/cleanhop/`) is used to remove potential misassigned reads due to index hopping. It subtracts to the number of reads, for each cell and for each gene, a percentage (0.5% by default) of the sum of the reads associated with the gene among all the cells sharing the same i7 index (column), and the sum of all the reads associated with the gene among all the cells sharing the same i5 index (row). Finally, read counts are normalized by counts per million.

B Solver description

The main principle of the scCross solver is to use a beam search to efficiently find a first approximate solution, which serves as initialization to a local search algorithm. The beam search is modified to take advantage of the sample identity of each cell, so as to ensure diversity in the search and avoid getting stuck in a region of the search space that is relative to only one sample. Indeed, an optimal solution to the scCross optimization problem is expected to contain many cells from different samples. However, the beam search starts from clusters containing only a few cells. Therefore, the δ_i factor gives a large penalty to all solutions, canceling out its effect during this early phase. The beam search might thus be drawn to a search space region relative to only one sample and get stuck there.

The first adaptation consists in adding an extra initialization step, which aims to find a small solution present in as many samples as possible by combining pairs of cells from different samples. It starts by selecting the 100 best pairs of each sample, which form new surrogate variables for a search where only pairs from different samples are combined (i.e. solutions of this initialization step contain at most 2 cells from the same sample). The best solutions from this initialization step form the starting point of the actual beam search, which explores the search space level by level by adding one cell to solutions from the previous level.

While the initialization step guarantees that the beam search starts with a cluster of cells coming from at least two different samples, the beam search could in some cases only add cells coming from one sample. To avoid filling the beam only with solutions related to one sample, the solver maintains one beam for each sample during the first levels of the beam search. This ensures a larger diversity of solutions if the search is temporarily dominated by sample-specific ones. In the long run, cross-sample solutions will be favored thanks to the effect of δ_i, and all beams will tend to contain the same solutions (thus going back to a regular beam search).

Finally, the solution of the beam search serves as initialization for a simulated annealing algorithm, which will stochastically explore its neighborhood in order to improve the quality of the solution and produce the final scCross result.

Figure A1 shows the execution time of scCross evolves linearly with respect to the number of cells.

C Parameter analysis

Figures A2 to A4 show the influence of the values of the κ and μ parameters on the number of cells and genes included in the scCross solution for the cases studies reported in sections 3.3 to 3.5 of the main manuscript. The general trend that can be observed in these figures is that the number of genes in inversely correlated to

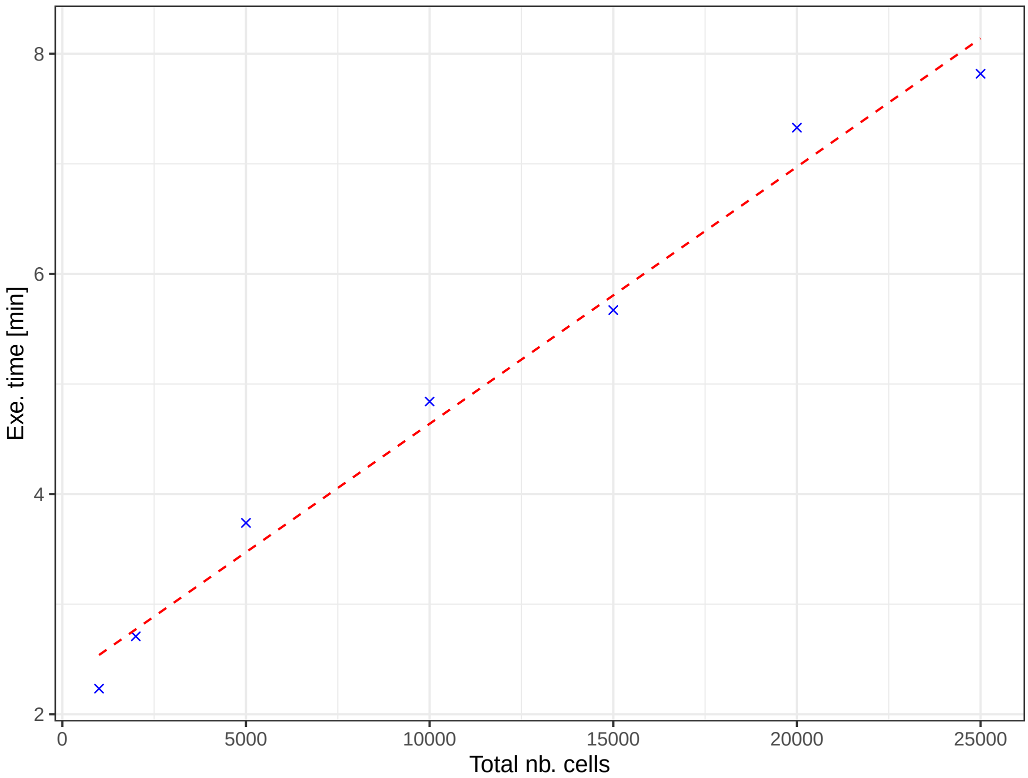

Figure A1: Execution time of scCross when sampling datasets of various sizes from the NSCLC DTCs dataset. This experiment was conducted on a MacBook Pro laptop (Mac OS 14.3.1, Apple M1 pro CPU, 16GB RAM).

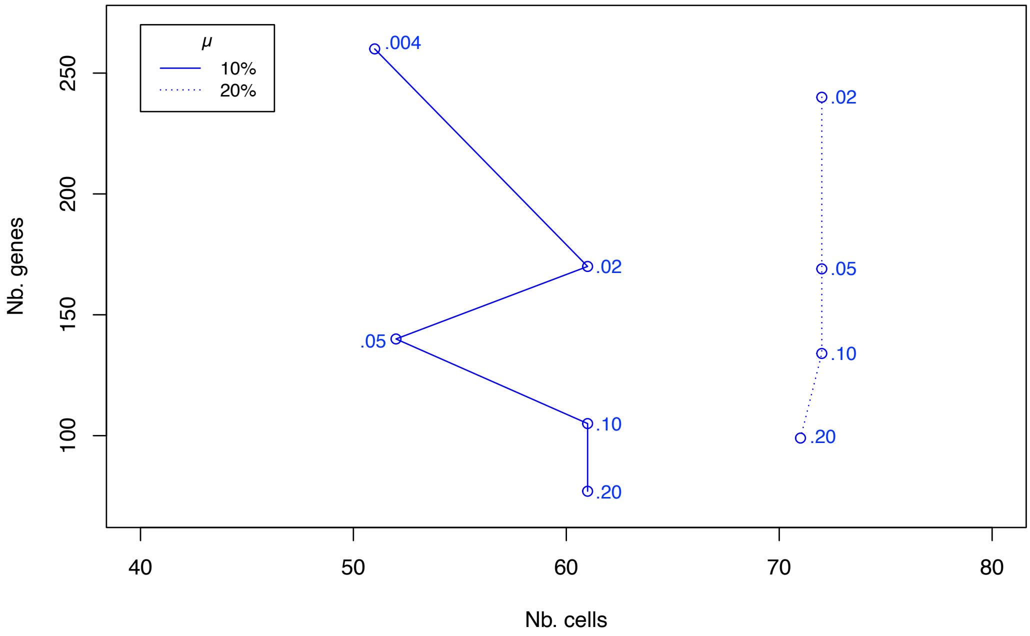

Figure A2: Number of cells and genes identified by scCross in the NSCLC DTCs data in function of the κ parameter (values indicated next to the dots), for two different values of the μ parameter.

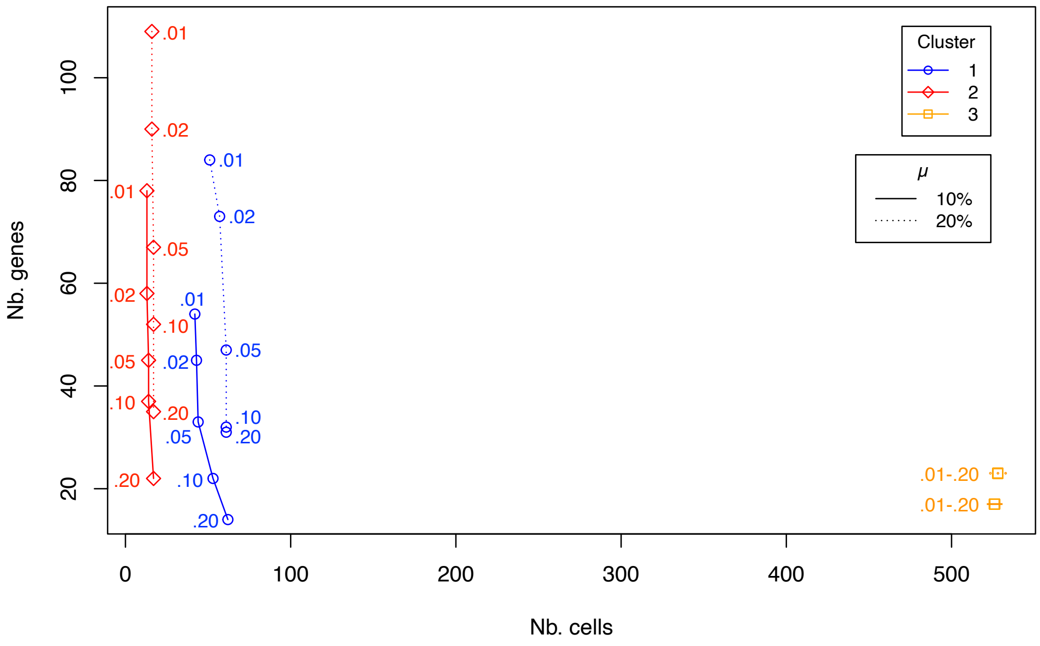

Figure A3: Number of cells and genes for the three biclusters identified by scCross in the pancreas data in function of the κ parameter, for two different values of the μ parameter.

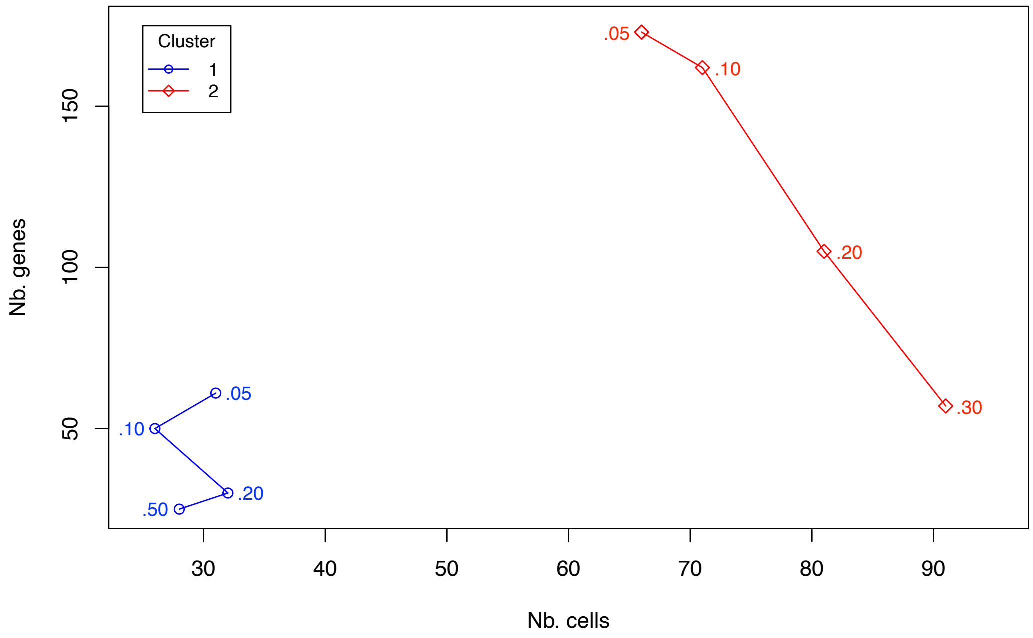

Figure A4: Number of cells and genes for the two biclusters identified by scCross in the NSCLC DTCs data in function of the κ parameter. The results are reported here for $\mu = 20\%$, as the default value of 10% yields a solution with only two genes (for the first bicluster). This indicates that the constraint on the maximum number of negative values allowed in the bicluster is probably too strict, motivating the fact to increase the value of μ.

the value of κ. Indeed, whenever κ is increased, the number of genes in the solution decreases. This makes sense given the definition of the scCross optimization problem, as it inflicts a larger penalty on out-of-cluster expression and therefore only keeps the most deferentially expressed genes.

One should note that tuning this κ parameter didn't change the biological interpretation of the biclusters in any of our experiments. One might therefore choose the value of κ based on the number of genes that is deemed appropriate for visualization purposes and/or biological interpretation (e.g. using a Gene Onthology analysis). For instance, we here chose to increase the value of κ whenever the number of genes returned with the default κ value is significantly higher than 60, so as to be able to display the entire bicluster in the figures below (A8 to A15). A Gene Onthology analysis was easily conducted with the obtained number of genes (ranging from 17 to 77 genes).

The μ parameter controls the maximum proportion of negative values that is allowed within the bicluster. By default, it is set to 10%. Increasing this parameter (for instance to 20%) naturally leads to more cells and genes being included in the solution. Indeed, the number of genes increases as genes that contained more negative values (e.g. have higher dropout rates) can now be included. We therefore suggest to increase the value of μ whenever the number of genes returned with $\mu = 10\%$ is very low (the *tabula muris* case study gives an example as only two genes are included in the solution). Increasing μ also leads to an increase in the number of cells, as cells that express a significant part of the genes identified with $\mu = 10\%$ may now be included. We therefore suggest to increase μ whenever a visual inspection of the results shows that some out-of-cluster cells express a significant part (for instance 2/3) of the marker genes.

D Gene prefiltering

The focus of the scCross method is to identify rare subpopulations of cells shared across different samples. However, the scCross optimization problem (equations (1)–(4) in the main manuscript) could in principle identify a large cell population whenever many genes are expressed in nearly all cells. In the limit, all cells could be part of the selected bicluster ($J = \mathcal{C}$) and the second term of equation (1) would vanish. scCross therefore filters out initially any gene expressed in more than $x\%$ of the cells. This initial filtering is motivated by the search of gene markers of specific subpopulations rather than generic markers of high expression throughout the cell population.

Such a threshold makes sense as the the distribution of genes, in terms of the number of cells they are expressed in, generally follows a power law (figure A5). One typically sets a threshold corresponding to the beginning of the long tail of the distribution. By default, a threshold of $x = 25\%$ of the cells is used, which yields good results in all of our experiments as the vast majority of genes clear this bar. Yet, other thresholds could be considered.

Figure A5a shows the genes kept after a 25%, a 33%, and a 50% threshold on the GARP+ Tregs data (see section 3.1 of the main manuscript). Table A1 reports the results of scCross after applying the different thresholds. The same subpopulation of 31 cells is identified in all cases. Only the number of genes vary as some genes that didn't pass the initial filtering are included when increasing the threshold. Yet, these genes have little influence in the solution: the result after a 50% threshold contains 60% more genes than the one obtained after a 25% filtering, but the objective value is increased by only 7%. This makes sense as these genes will have more out-of-cluster expression, which will be penalized by the second term of equation (1) and lead to a low contribution to the objective value. Changing the filtering threshold therefore has little influence on the identified cell subpopulation.

Rather than using a fixed threshold, one could take advantage of the multiple run strategy to eliminate large biclusters. Indeed, a first run of scCross could be used to first identify any large bicluster present in the data. One would then filter out the corresponding genes, which are representative of this large subpopulation of cells, to perform a second run of scCross to search for a rare subpopulation of cells. In the limit, one could relax the constraint on the proportion of negative values allowed in the bicluster by setting $\mu = 100\%$ in this first run to identify the largest possible bicluster. This would be analog to the max-sum submatrix approach (Branders *et al.*, 2019), whose purpose is precisely to identify large biclusters of cells and genes. On the GARP+ Tregs data, this leads to a bicluster containing 4047 genes and all cells. Removing these genes roughly corresponds to a 33% threshold, both in terms of the genes kept (figure A5b) and the subsequent scCross result (fourth line of table A1). Given the computational overhead of performing multiple runs, we favor the pragmatic approach of defining a fixed threshold.

As an alternative to simply filtering out genes, one could use other types of data normalization to search for other kinds of patterns. For instance, considering the opposite of the original expression values would lead to select genes that are underexpressed in the selected cells. Other data normalizations can also lead to other interesting patterns (e.g. the genes departing the most from the median expression values for each cell). We evaluate the first approach on the GARP+ Tregs data by inverting the sign of the expression values of all genes expressed in more than 50% of the cells. Yet, this results in the exact same solution as the one obtained when

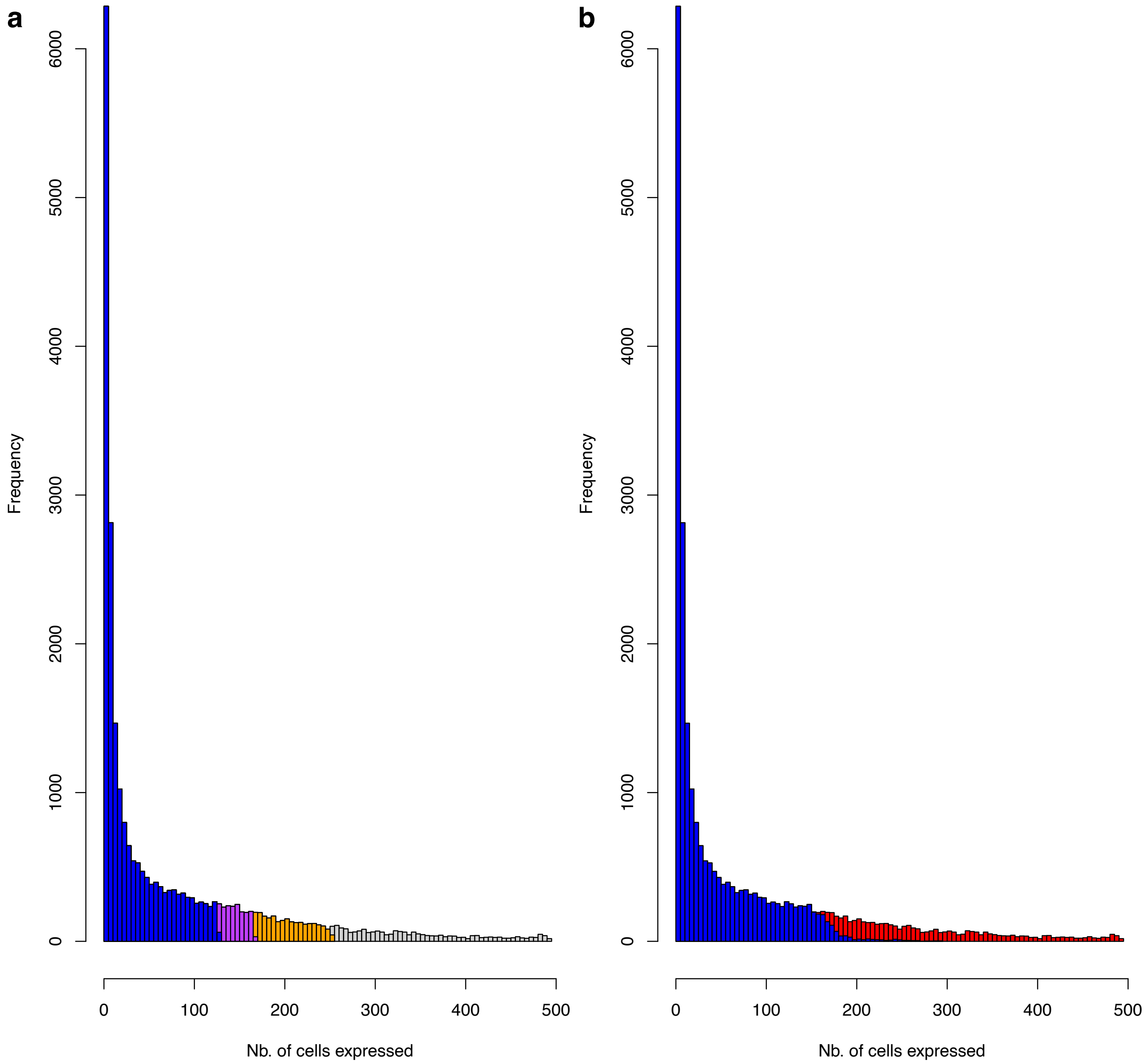

Figure A5: Histogram of the genes, in function of the number of cells they are expressed in, for the GARP+ Tregs data. Only genes expressed in at least two different cells are considered. **a** Genes kept after a 25% (blue), 33% (purple), and a 50% (orange) threshold. **b** In red, the genes forming part of the scCross solution with $\mu = 100\%$, which would be removed in subsequent runs to identify rare subpopulations of cells.

Table A1: Results of scCross on the GARP+ Tregs data depending on the chosen preprocessing.

Reprocessing	Genes after filt.	Cells in sol.	Genes in sol.	Objective val.	
Cutoff 25%	17 250	31	60	1019	
Cutoff 33%	19 017	31	78	1067	(+ 4.7%)
Cutoff 50%	21 370	31	96	1092	(+ 7.1%)
Remove 1st run	21 988	31	81	1065	(+ 4.5%)
Opposite > 50%	26 035	31	96	1092	(+ 7.1%)

simply removing those genes.

E Gene Onthology functions

Tables A2 to A7 report the results of the Gene Ontology analysis for the subpopulations identified by scCross in the different case studies. This analysis has been performed using the `clusterProfiler` R package. For each one, the 20 most significantly enriched GO terms are reported. A functional interpretation of these terms is included in the main manuscript.

F Cilia Carta genes

Tables A8 and A8 indicate which of the genes characterizing the cilium subpopulation identified in the NSCLC DTCs data have been identified in the *Cilia Carta* compendium.

G Additional heatmaps

This section contains several heatmaps which augment the ones present in the main manuscript.

Section 3.1: breast cancer GARP+ Treg data

- **Figure A6** corresponds to **Figure 3b** in the main manuscript, which only presents genes associated to the 8th bicluster found by MicroCellClust. Figure A6 shows 10 genes for each cluster identified by MicroCellClust.
- **Figure A7** corresponds to **Figure 3c** of the main manuscript. It shows the result of scCross with all selected genes displayed.

Section 3.3: non-small cell lung cancer dissociated tumor cells data

- **Figure A8** corresponds to **Figure 4a** of the main manuscript. It show the result of scCross with all selected genes displayed.
- **Figure A9** corresponds to **Figure 4b** of the main manuscript. It show the result of scCross on the MNN integrated data.

Section 3.4: pancreas data

- **Figure A10** corresponds to the first cluster displayed in **Figure 5** of the main manuscript.
- **Figure A11** corresponds to the second cluster displayed in **Figure 5** of the main manuscript.
- **Figure A12** corresponds to the third cluster displayed in **Figure 5** of the main manuscript.

Section 3.5: *tabula muris*

- **Figure A13** corresponds to the first cluster displayed in **Figure 6** of the main manuscript.
- **Figure A14** shows the second result of scCross when using the same parameters ($\kappa = 0.044$ and $\mu = 0.2$) as for the first result (Figure A13).
- **Figure A15** shows the second result of scCross with $\kappa = 0.5$, which corresponds to the second cluster of **Figure 6** of the main manuscript.

Table A2: GO terms for the subpopulation identified in the NSCLC DTCs data (section 3.3 of the main manuscript).

	ID	Description	p.adjust	GeneRatio
1	GO:0003341	cilium movement	5.0524e-22	17/38
2	GO:0007018	microtubule-based movement	1.7572e-21	20/38
3	GO:0035082	axoneme assembly	2.5309e-17	12/38
4	GO:0001578	microtubule bundle formation	5.5063e-16	12/38
5	GO:0060294	cilium movement involved in cell motility	3.6587e-15	12/38
6	GO:0001539	cilium or flagellum-dependent cell motility	5.8895e-15	12/38
7	GO:0060285	cilium-dependent cell motility	5.8895e-15	12/38
8	GO:0044782	cilium organization	1.8517e-14	15/38
9	GO:0060271	cilium assembly	1.7246e-13	14/38
10	GO:0030317	flagellated sperm motility	9.3987e-11	9/38
11	GO:0097722	sperm motility	9.3987e-11	9/38
12	GO:0044458	motile cilium assembly	9.2067e-10	7/38
13	GO:0003351	epithelial cilium movement involved in extracellular fluid movement	3.6916e-09	6/38
14	GO:0006858	extracellular transport	5.3579e-09	6/38
15	GO:0036159	inner dynein arm assembly	5.4005e-07	4/38
16	GO:0007286	spermatid development	1.8768e-06	7/38
17	GO:0048515	spermatid differentiation	2.2408e-06	7/38
18	GO:0007288	sperm axoneme assembly	3.8099e-06	4/38
19	GO:0120316	sperm flagellum assembly	1.2823e-05	4/38
20	GO:0003352	regulation of cilium movement	1.4323e-05	4/38

Table A3: GO terms for the first subpopulation identified in the pancreas data (section 3.4 of the main manuscript).

	ID	Description	p.adjust	GeneRatio
1	GO:0042119	neutrophil activation	1.0262e-06	5/22
2	GO:0036230	granulocyte activation	1.0281e-06	5/22
3	GO:0002283	neutrophil activation involved in immune response	1.6384e-06	4/22
4	GO:0002697	regulation of immune effector process	1.6611e-06	8/22
5	GO:0002443	leukocyte mediated immunity	1.8636e-06	8/22
6	GO:0002366	leukocyte activation involved in immune response	4.5741e-06	7/22
7	GO:0002263	cell activation involved in immune response	4.5741e-06	7/22
8	GO:0002683	negative regulation of immune system process	5.2637e-06	8/22
9	GO:0002275	myeloid cell activation involved in immune response	8.5849e-06	5/22
10	GO:0002695	negative regulation of leukocyte activation	8.5849e-06	6/22
11	GO:0050866	negative regulation of cell activation	1.4766e-05	6/22
12	GO:1902563	regulation of neutrophil activation	4.6081e-05	3/22
13	GO:0002886	regulation of myeloid leukocyte mediated immunity	4.6081e-05	4/22
14	GO:0051250	negative regulation of lymphocyte activation	8.2199e-05	5/22
15	GO:0002888	positive regulation of myeloid leukocyte mediated immunity	1.0990e-04	3/22
16	GO:0032928	regulation of superoxide anion generation	1.1921e-04	3/22
17	GO:0022408	negative regulation of cell-cell adhesion	1.7998e-04	5/22
18	GO:0002274	myeloid leukocyte activation	3.2344e-04	5/22
19	GO:0097529	myeloid leukocyte migration	3.2344e-04	5/22
20	GO:0002444	myeloid leukocyte mediated immunity	3.2344e-04	4/22

Table A4: GO terms for the second subpopulation identified in the pancreas data (section 3.4 of the main manuscript).

	ID	Description	p.adjust	GeneRatio
1	GO:0000280	nuclear division	5.6920e-27	23/34
2	GO:0048285	organelle fission	2.8744e-26	23/34
3	GO:0007059	chromosome segregation	5.4023e-24	21/34
4	GO:0098813	nuclear chromosome segregation	1.3084e-22	19/34
5	GO:0140014	mitotic nuclear division	3.1803e-22	18/34
6	GO:0000819	sister chromatid segregation	1.8671e-21	17/34
7	GO:0000070	mitotic sister chromatid segregation	2.3947e-19	15/34
8	GO:0044772	mitotic cell cycle phase transition	1.4433e-18	18/34
9	GO:0051983	regulation of chromosome segregation	3.1870e-16	12/34
10	GO:0030071	regulation of mitotic metaphase/anaphase transition	3.7732e-14	10/34
11	GO:1902099	regulation of metaphase/anaphase transition of cell cycle	4.8086e-14	10/34
12	GO:0007091	metaphase/anaphase transition of mitotic cell cycle	4.9206e-14	10/34
13	GO:0051783	regulation of nuclear division	5.5664e-14	11/34
14	GO:0044784	metaphase/anaphase transition of cell cycle	5.8252e-14	10/34
15	GO:0033045	regulation of sister chromatid segregation	1.0061e-13	10/34
16	GO:1905818	regulation of chromosome separation	2.6983e-13	9/34
17	GO:0007088	regulation of mitotic nuclear division	2.9875e-13	10/34
18	GO:0033044	regulation of chromosome organization	4.0758e-13	12/34
19	GO:0007094	mitotic spindle assembly checkpoint signaling	4.0758e-13	8/34
20	GO:0071173	spindle assembly checkpoint signaling	4.0758e-13	8/34

Table A5: GO terms for the third subpopulation identified in the pancreas data (section 3.4 of the main manuscript).

	ID	Description	p.adjust	GeneRatio
1	GO:0030199	collagen fibril organization	6.3798e-09	6/16
2	GO:0030198	extracellular matrix organization	6.1434e-07	7/16
3	GO:0043062	extracellular structure organization	6.1434e-07	7/16
4	GO:0045229	external encapsulating structure organization	6.1434e-07	7/16
5	GO:0071230	cellular response to amino acid stimulus	6.8507e-07	5/16
6	GO:0071229	cellular response to acid chemical	9.8134e-07	5/16
7	GO:0043200	response to amino acid	3.8033e-06	5/16
8	GO:0001101	response to acid chemical	6.2356e-06	5/16
9	GO:0060348	bone development	8.3899e-05	5/16
10	GO:0032963	collagen metabolic process	1.1646e-04	4/16
11	GO:0061448	connective tissue development	1.4692e-04	5/16
12	GO:0071711	basement membrane organization	1.4692e-04	3/16
13	GO:0085029	extracellular matrix assembly	2.8402e-04	3/16
14	GO:0032964	collagen biosynthetic process	4.7617e-04	3/16
15	GO:0060350	endochondral bone morphogenesis	7.2685e-04	3/16
16	GO:0051216	cartilage development	8.8808e-04	4/16
17	GO:0038063	collagen-activated tyrosine kinase receptor signaling pathway	1.4056e-03	2/16
18	GO:0043589	skin morphogenesis	1.5922e-03	2/16
19	GO:0060349	bone morphogenesis	2.5769e-03	3/16
20	GO:0038065	collagen-activated signaling pathway	2.6001e-03	2/16

Table A6: GO terms for the first subpopulation identified in the *tabula muris* data (section 3.5 of the main manuscript).

	ID	Description	p.adjust	GeneRatio
1	GO:0002443	leukocyte mediated immunity	3.0556e-11	15/56
2	GO:0002444	myeloid leukocyte mediated immunity	3.5094e-11	10/56
3	GO:0002274	myeloid leukocyte activation	7.1583e-11	12/56
4	GO:0002275	myeloid cell activation involved in immune response	3.3488e-10	9/56
5	GO:0043299	leukocyte degranulation	2.0241e-09	8/56
6	GO:0045576	mast cell activation	2.0419e-09	8/56
7	GO:0043303	mast cell degranulation	5.0292e-09	7/56
8	GO:0002366	leukocyte activation involved in immune response	5.0292e-09	11/56
9	GO:0002448	mast cell mediated immunity	5.0292e-09	7/56
10	GO:0002263	cell activation involved in immune response	5.0292e-09	11/56
11	GO:0002279	mast cell activation involved in immune response	5.0292e-09	7/56
12	GO:0045055	regulated exocytosis	9.7862e-09	10/56
13	GO:0002886	regulation of myeloid leukocyte mediated immunity	2.0849e-08	7/56
14	GO:0006887	exocytosis	2.4006e-08	11/56
15	GO:0032418	lysosome localization	6.1318e-08	7/56
16	GO:1990849	vacuolar localization	6.1318e-08	7/56
17	GO:0050727	regulation of inflammatory response	3.1718e-07	10/56
18	GO:0002703	regulation of leukocyte mediated immunity	9.1296e-07	9/56
19	GO:0043304	regulation of mast cell degranulation	1.1063e-06	5/56
20	GO:0050866	negative regulation of cell activation	1.3120e-06	8/56

Table A7: GO terms for the second subpopulation identified in the *tabula muris* data (section 3.5 of the main manuscript).

	ID	Description	p.adjust	GeneRatio
1	GO:0007059	chromosome segregation	9.5400e-50	35/52
2	GO:0000819	sister chromatid segregation	2.0976e-48	30/52
3	GO:0098813	nuclear chromosome segregation	5.3012e-48	32/52
4	GO:0000070	mitotic sister chromatid segregation	7.3412e-47	28/52
5	GO:0140014	mitotic nuclear division	5.2846e-46	30/52
6	GO:0000280	nuclear division	4.9782e-43	32/52
7	GO:0051983	regulation of chromosome segregation	2.7251e-41	24/52
8	GO:0051304	chromosome separation	7.0365e-39	21/52
9	GO:1905818	regulation of chromosome separation	1.0509e-37	20/52
10	GO:0007088	regulation of mitotic nuclear division	4.8092e-31	19/52
11	GO:0033044	regulation of chromosome organization	1.2085e-30	22/52
12	GO:0033047	regulation of mitotic sister chromatid segregation	3.8918e-30	16/52
13	GO:0033045	regulation of sister chromatid segregation	5.9684e-30	18/52
14	GO:0010965	regulation of mitotic sister chromatid separation	5.9684e-30	16/52
15	GO:0044772	mitotic cell cycle phase transition	5.9684e-30	25/52
16	GO:0051306	mitotic sister chromatid separation	1.3296e-29	16/52
17	GO:0051783	regulation of nuclear division	4.5944e-29	19/52
18	GO:0033046	negative regulation of sister chromatid segregation	6.9632e-29	15/52
19	GO:0033048	negative regulation of mitotic sister chromatid segregation	6.9632e-29	15/52
20	GO:0045841	negative regulation of mitotic metaphase/anaphase transition	6.9632e-29	15/52

Table A8: Cilia Carta data for the subpopulation identified in the NSCLC DTCs data (section 3.3 of the main manuscript). This table continues on page 11.

Gene Name	CiliaCarta Rank	CiliaCarta Score	Gene Onthology	Gold standard
C20orf85	122	4.348476959		
C11orf88	212	2.943095083		
ENKUR	54	6.472108308	True	
DYNLRB2	18	7.475605752	True	
C1orf194	131	4.348476959		
ZMYND10	140	4.063228988	True	
CFAP53				
EFCAB1	118	4.348476959		
C9orf24	325	1.939597639		
TMEM190				
MORN5	334	1.939597639		
ERICH3				
CAPSL	208	2.943095083		
CFAP126				
CDHR3	206	2.943095083		
SAXO2				
C5orf49	132	4.348476959		
DNAH6	109	4.63427188	True	True
FAM216B	333	1.939597639		
C9orf135	338	1.939597639		
WDR38				
ANKRD66				
SNTN	336	1.939597639	True	
TSPAN19				
CFAP43				
FAM92B	3702	-3.974226523	True	
CCDC78	322	1.939597639		
FAM81B	209	2.943095083		
LRRC46	125	4.348476959		
LDLRAD1				
AL357093.2				
AC013264.1				
C6orf118	120	4.348476959		
TEKT1	326	1.939597639	True	
ZBBX	328	1.939597639		
MS4A8				
ROPN1L	144	4.063228988	True	True
DYDC2	123	4.348476959		
ARMC3	72	5.351974403		
RSPH9	87	5.066726433	True	True
CCDC39	595	0.144718304	True	True
CASC1	313	1.939597639		
TUBA4B				
PPP1R42				
CABCOCO1				
ARMC4	539	0.49832414	True	
RSPH4A	173	3.673731529	True	True
RIIAD1				
DNAH12	112	4.63427188	True	
LRRC10B				
SPATA18				
RP1	504	0.74932291	True	True

Table A8: Cilia Carta data for the subpopulation identified in the NSCLC DTCs data (section 3.3 of the main manuscript). This table starts on page 10.

Gene Name	CiliaCarta Rank	CiliaCarta Score	Gene Onthology	Gold standard
C11orf97				
C22orf15				
ECRG4				
C7orf57	324	1.939597639		
STMND1				
RIBC1	44	7.0431512		
CCL15	321	1.939597639		
CFAP52				
EFCAB10				
CFAP73				
CD164L2				
TEKT2	93	4.919519851	True	True
SRGAP3-AS2				
AKAP14	335	1.939597639	True	
DNAH9	14	7.475605752	True	
OMG				
CFAP299				
CFAP206				
PACRG	39	7.0431512	True	True
SPAG6	81	5.066726433	True	True
PRR29				
CFAP157				
SPEF1	83	5.066726433	True	
CCDC17	320	1.939597639		
UBXN10	159	3.916022407	True	

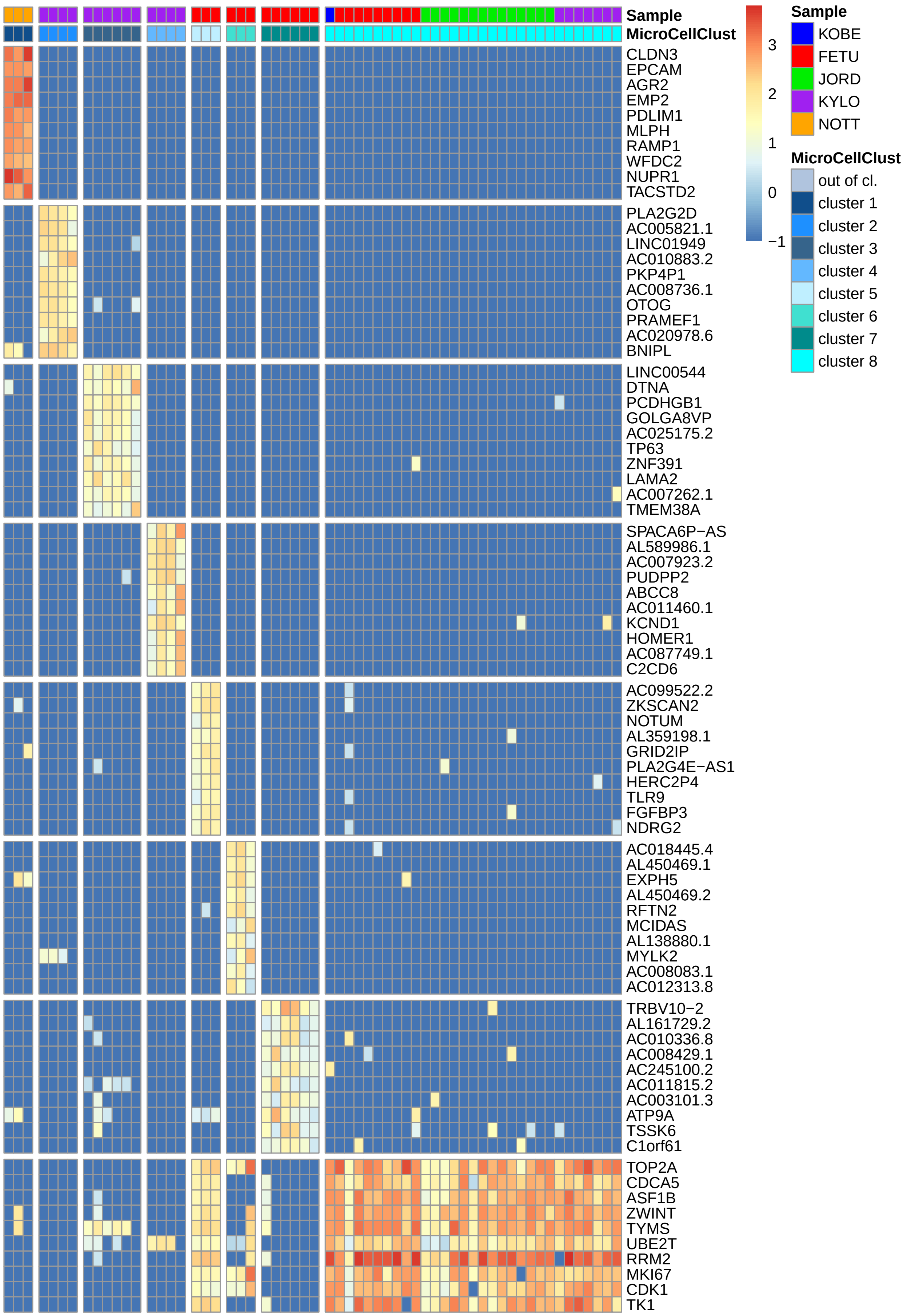

Figure A6: 10 genes with highest contribution to the objective for each cluster returned by MicroCellClust on the breast cancer data. Out of cluster cells are not displayed.

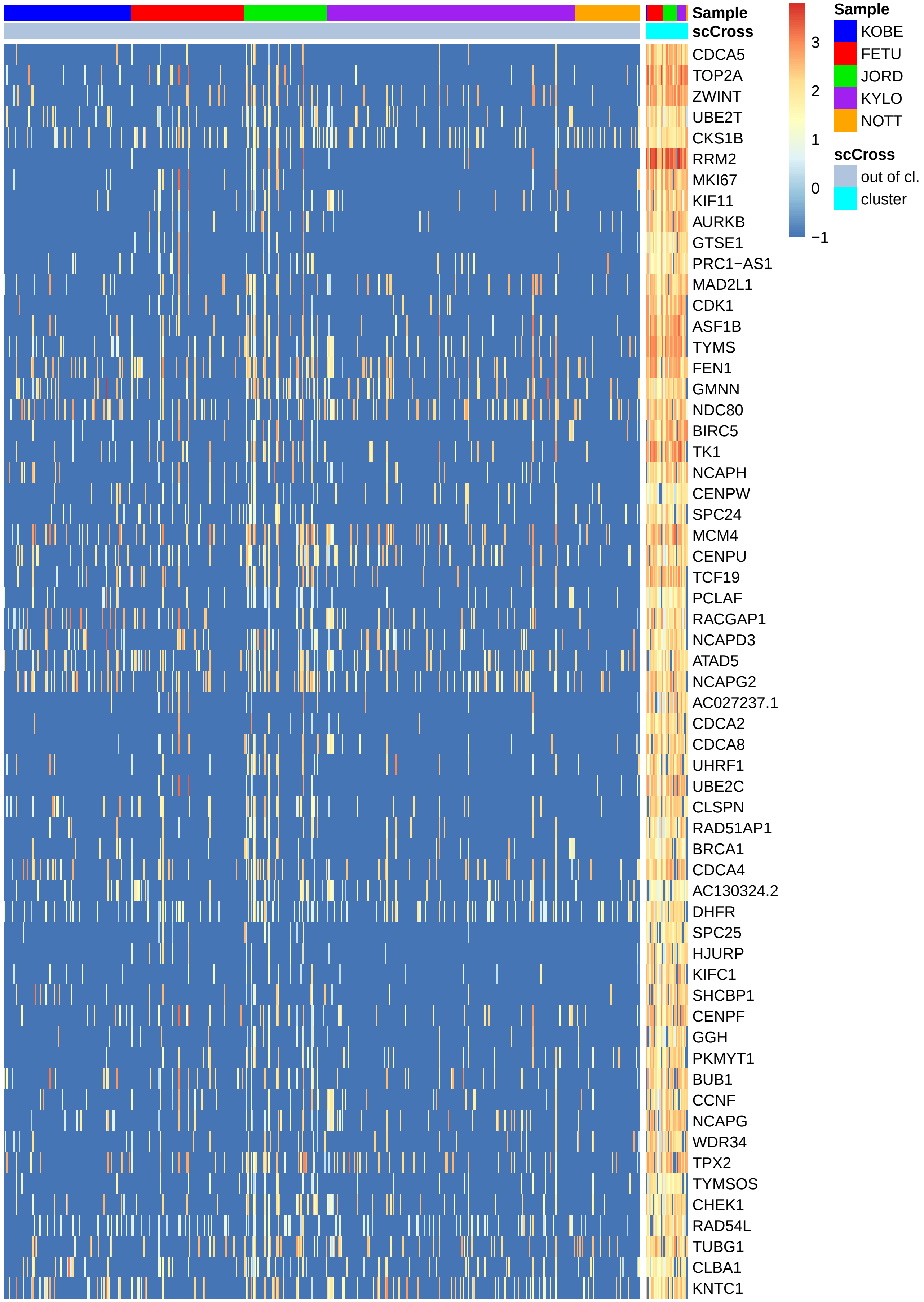

Figure A7: scCross result on the breast cancer data.

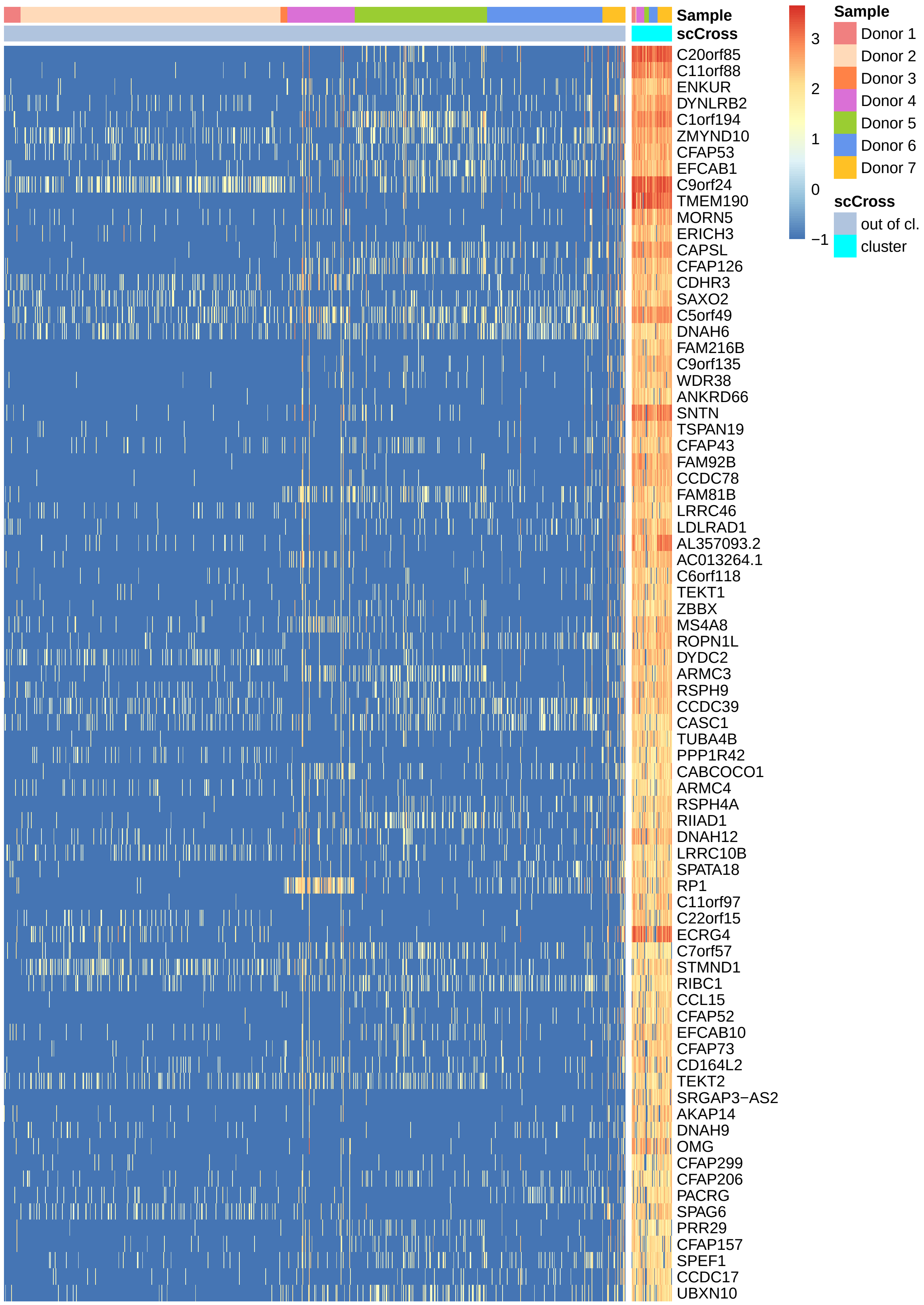

Figure A8: scCross result on the NSCLC DTCs dataset. Only the 1000 cells with highest objective value are displayed (out of 26 027).

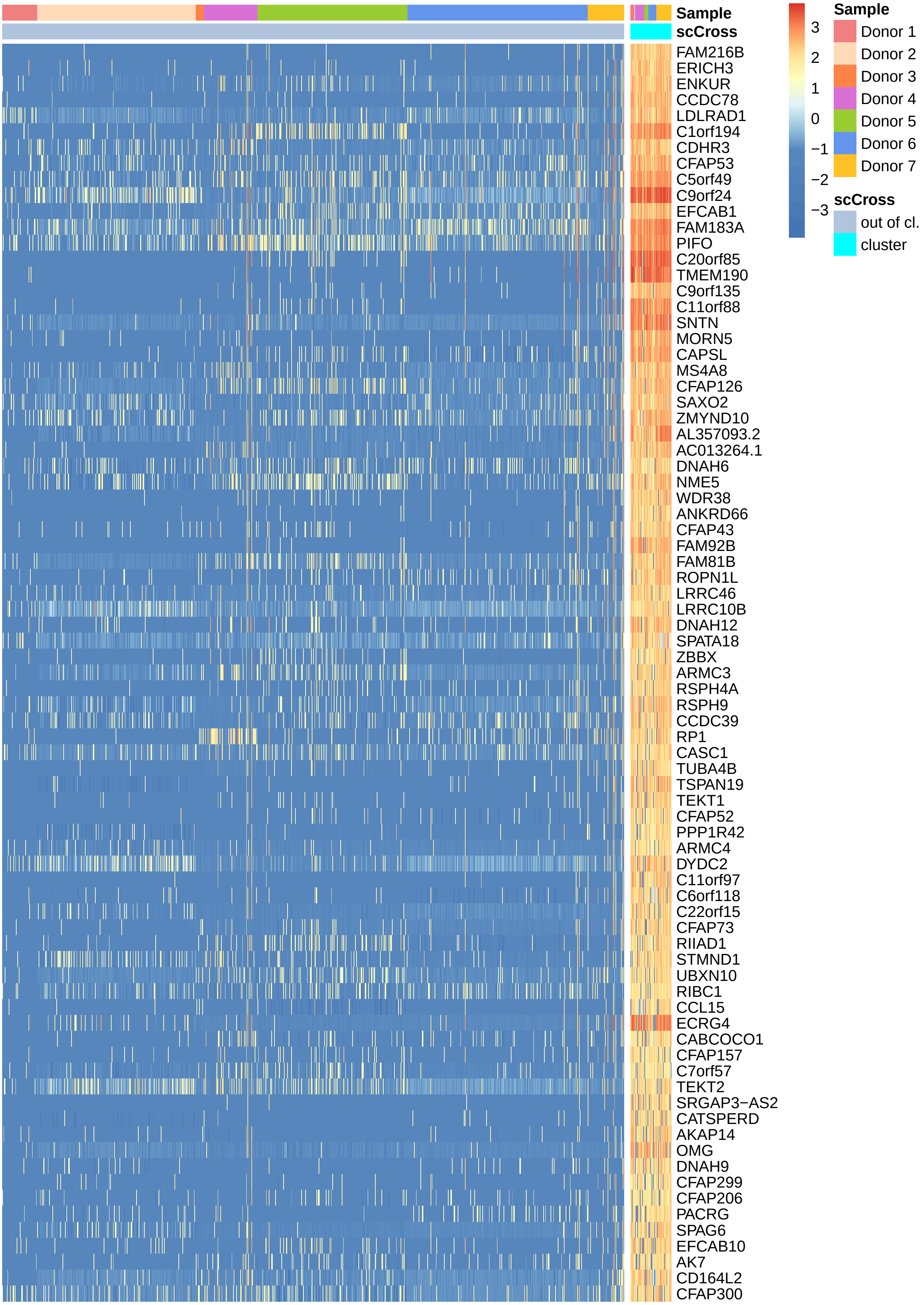

Figure A9: scCross result on the NSCLC DTCs dataset after MNN integration. Only the 1000 cells with highest objective value are displayed (out of 26 027).

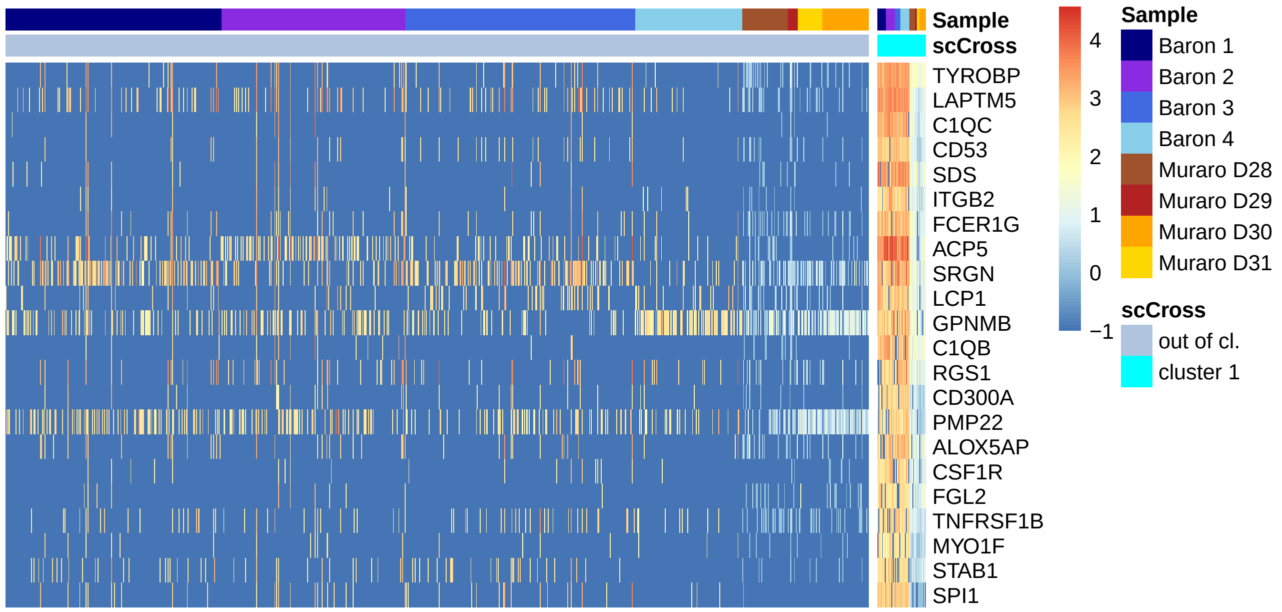

Figure A10: scCross result on the pancreas data. Only the 1000 cells with highest objective value are displayed (out of 11 641).

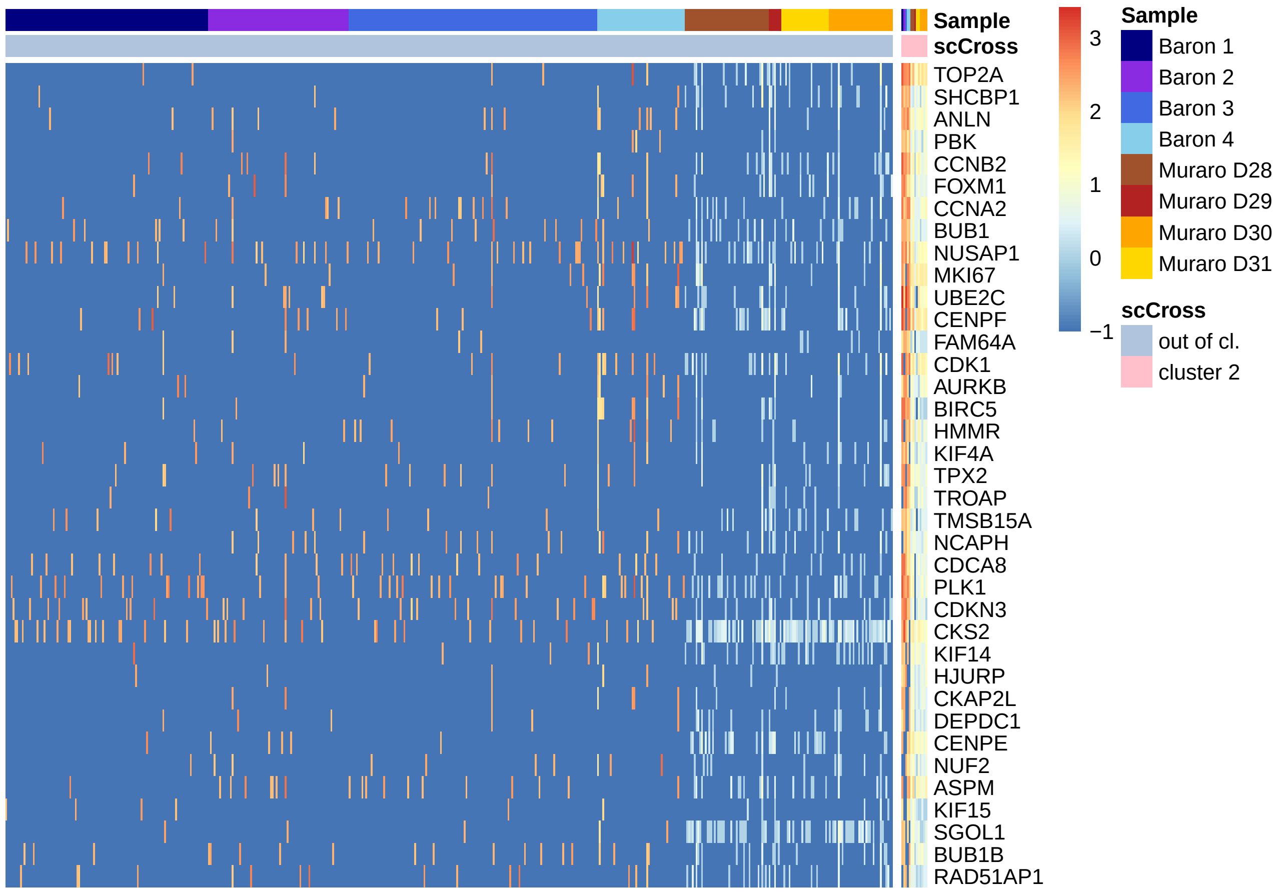

Figure A11: Second result of scCross on the pancreas data. Only the 500 cells with highest objective value are displayed (out of 11 641).

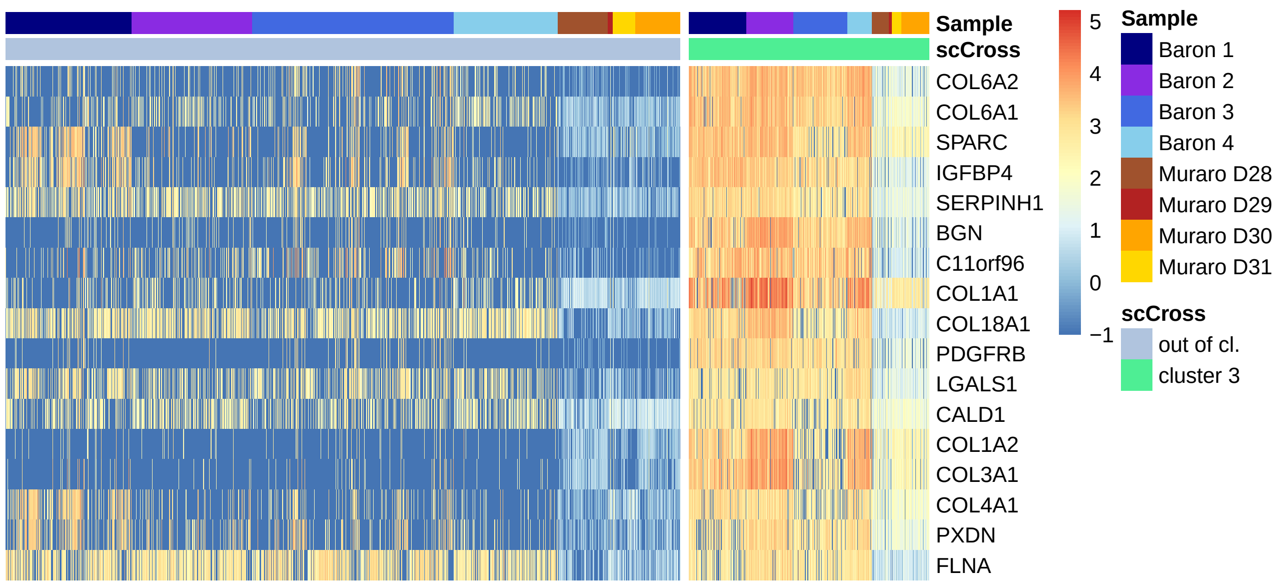

Figure A12: Third result of scCross on the pancreas data. Only the 2000 cells with highest objective value are displayed (out of 11 641).

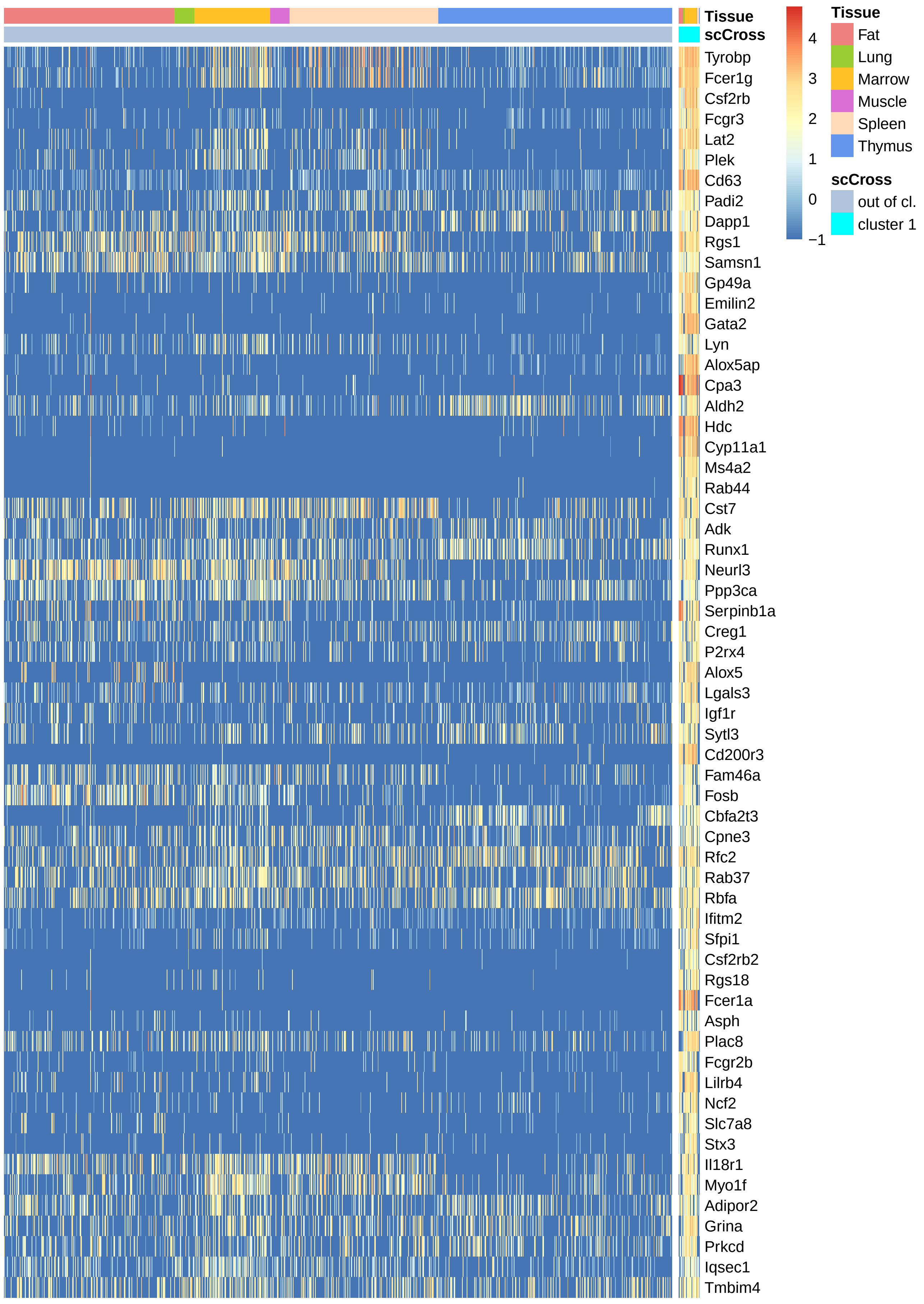

Figure A13: scCross result on the *Tabula Muris* data. Only the 1000 cells with highest objective value are displayed (out of 2251). The value of μ was set to 20%, as the default value of 10% yields a solutions with only two genes (Tyrobp and Fcer1g), which motivates the fact to relax the constraint on the maximum number of negatives values allowed within the bicluster.

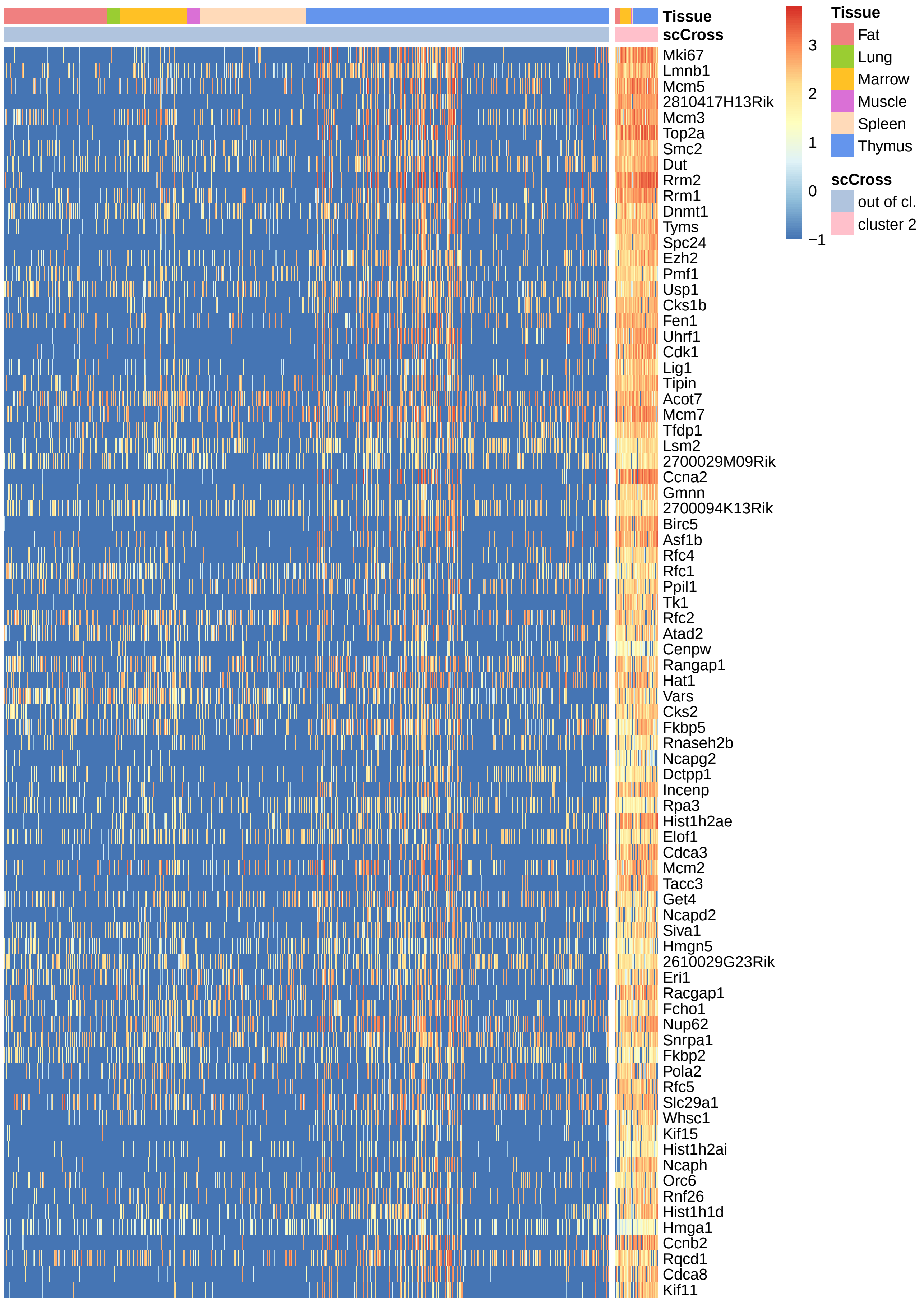

Figure A14: Second result of scCross on the *Tabula Muris* data. Only the 1000 cells with highest objective value are displayed (out of 2251), and 80 genes out of 173. The relatively large number of genes, as well as the fact that a lot of out of cluster cells express a significant part of the selected genes, motivates the fact to re-run scCross with an increased value for κ (see figure A15).

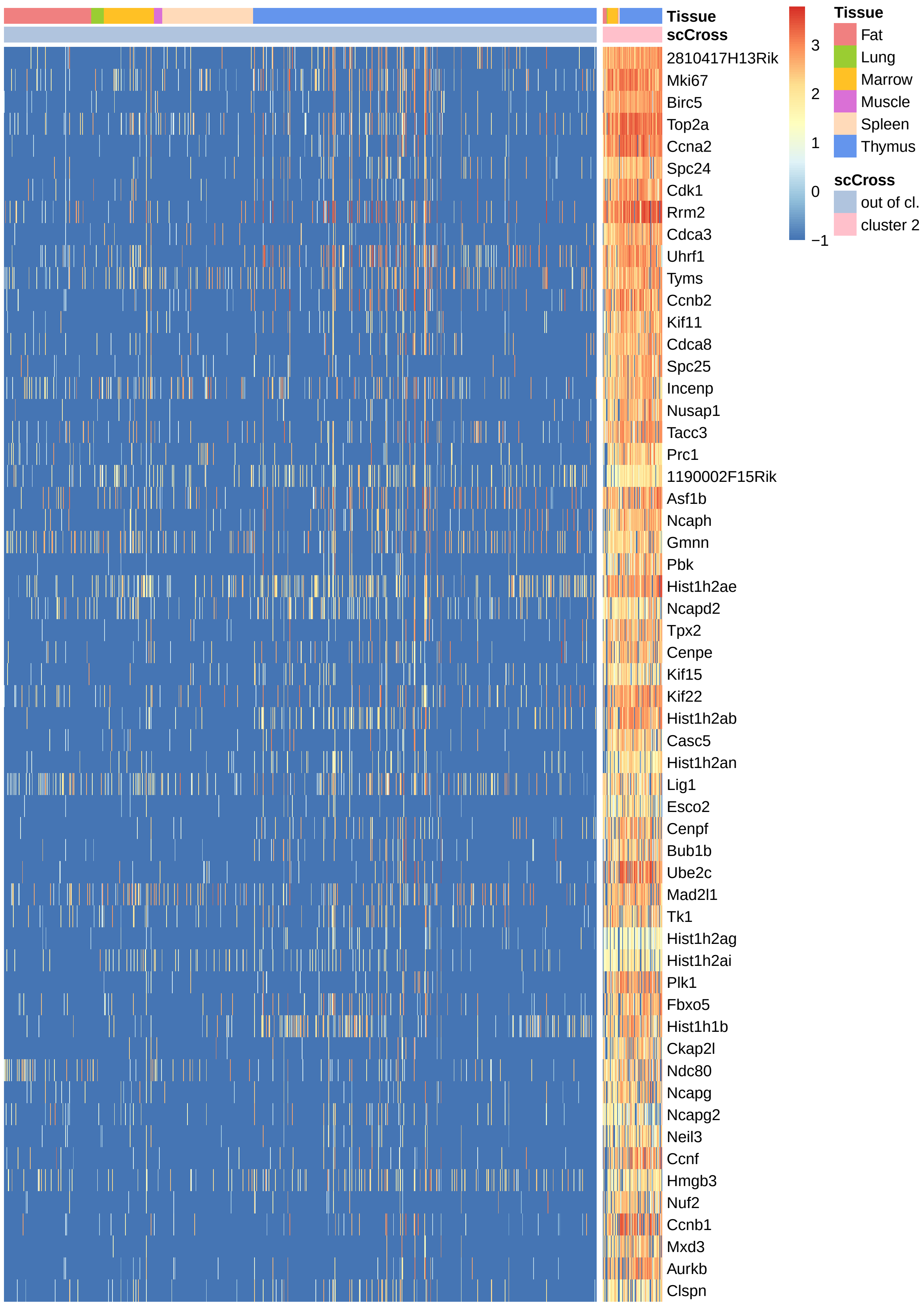

Figure A15: Second result of scCross on the *Tabula Muris* data with $\kappa = 0.5$ (obtained by taking the previous result and re-running only the local search with modified parameters). The increased value of κ leads to a stricter selection of genes (62 instead of 173), which in turn leads to the inclusion of 20 previously out of cluster cells.
